# Hybrid Silicon Nanowire Devices and Their Functional Diversity

**DOI:** 10.1002/advs.201900522

**Published:** 2019-06-03

**Authors:** Larysa Baraban, Bergoi Ibarlucea, Eunhye Baek, Gianaurelio Cuniberti

**Affiliations:** ^1^ Max Bergmann Center of Biomaterials and Institute for Materials Science Technische Universität Dresden 01062 Dresden Germany; ^2^ Center for Advancing Electronics Dresden (CfAED) TU Dresden 01062 Dresden Germany

**Keywords:** biosensors, field‐effect transistors, neuromorphics, photodetectors, silicon nanowires

## Abstract

In the pool of nanostructured materials, silicon nanostructures are known as conventionally used building blocks of commercially available electronic devices. Their application areas span from miniaturized elements of devices and circuits to ultrasensitive biosensors for diagnostics. In this Review, the current trends in the developments of silicon nanowire‐based devices are summarized, and their functionalities, novel architectures, and applications are discussed from the point of view of analog electronics, arisen from the ability of (bio)chemical gating of the carrier channel. Hybrid nanowire‐based devices are introduced and described as systems decorated by, e.g., organic complexes (biomolecules, polymers, and organic films), aimed to substantially extend their functionality, compared to traditional systems. Their functional diversity is explored considering their architecture as well as areas of their applications, outlining several groups of devices that benefit from the coatings. The first group is the biosensors that are able to represent label‐free assays thanks to the attached biological receptors. The second group is represented by devices for optoelectronics that acquire higher optical sensitivity or efficiency due to the specific photosensitive decoration of the nanowires. Finally, the so‐called new bioinspired neuromorphic devices are shown, which are aimed to mimic the functions of the biological cells, e.g., neurons and synapses.

## 1 Introduction

Advances in micro‐ and nanofabrication technologies have dramatically improved the quality and performance of the electronic devices, while reducing costs and overall dimensions. One of the prominent examples is the transistor that has faced a scalability evolution like no other element of the circuits: from the first demonstrated macroscale point‐contact germanium transistor in 1948 at Bell Laboratories^1^ to the billions of nanoscale ones fabricated in a single chip used for digital electronics. At the same time, the synergy of nanotechnology with chemistry and life sciences has opened a new route to use transistors, namely to track their analog signals, rather than using them only as a building block of logic gates.^2^, ^3^, ^4^, ^5^ Such an operation mode of semiconductor‐based nanodevices can be used, in particular, for the investigation of biochemical processes, bio‐ or photodetection, or bioinspired functionality with ultrahigh sensitivity. In this respect, the new generation of sensitive devices strongly benefit from surface effects, as the chemical or biological gating of, e.g., transistor sensors is a direct consequence of the change of the electrical surface potential *V*_surface_ at the nanoscaled transducer element. Namely, the change of the concentration of electrical charges in the microenvironment (caused, e.g., by the ions, molecules, or even cells), in a close proximity to the transducer, makes an enormous contribution to the modulation of channel conductivity.^6^ The highest sensitivity can be expected for semiconductor channels whose whole volume can be gated by surface charges,^7^, ^8^, ^9^ that is, below 50 nm.^8^ In these cases, the conductance of the whole cross section is dependent on the surrounding environment, responding with changes in charge carrier concentration to the variations in the nearby electric field.

**Figure 1:**
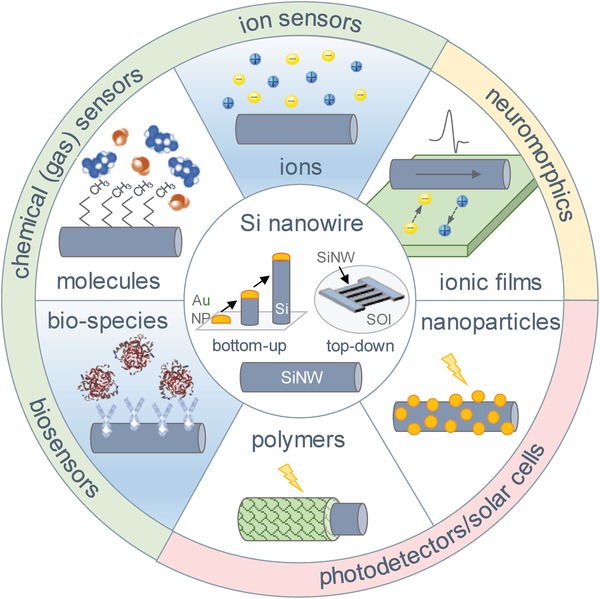
Various functionalities of SiNW devices. The central circle shows the fabrication methods of the SiNW. The second layer shows the different functionalized materials (organic or inorganic) on the SiNW. The outer layer shows their most common applications area.

**Figure 2:**
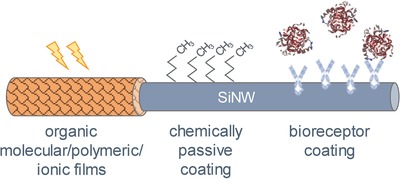
Organic and SiNW hybrid structures having different organic layers: an organic film coating using molecules, polymers, or ionic films for, e.g., photodetection or neuromorphic applications, a chemically passive coating (to prevent molecule adsorption or to be used as a reference sensor), and bioreceptor coating for specific analyte detection.

Apart from active participation in the progression of the new‐generation bio‐ and chemical sensors, the nanoscale elements also play a crucial role in the development of opto‐ and biomimetic electronics (see **Figure** **1**). Such a broad diversity of applications is frequently achieved thanks to the hybrid device architecture—smart combination of the conventional operation modes with the specific functional decoration of the electronic devices (see **Figure** **2**), e.g., molecular or polymeric coatings that surprisingly extend the functionality of the whole instrument.^10^, ^11^, ^12^, ^13^ Despite the enormous opportunities that hybrid nanodevices potentially offer to build new advantageous systems, the development of these systems still lies mostly in the research plane. There are a number of challenges at the moment that prohibit their rapid commercialization. Among them, we name the complex nature of interactions between organic (bio) species and nanoscale electronic matter,^14^, ^15^ as well as the need for integration and packaging of organic and inorganic subsystems on a single chip to reduce the cost.^16^, ^17^, ^18^, ^19^

During the last decade 1D nanostructures, and in particular semiconductor silicon nanowires (SiNWs), have attracted attention as highly efficient elements of transistor elements due to their high level of complementary metal‐oxide semiconductor (CMOS) compatibility,^20^ compactness of the resulting device architecture, and high surface‐to‐volume ratio. Indeed, in parallel to the SiNWs, strong efforts are dedicated to the implementation of other materials, e.g., graphene,^21^ molybdenum disulfide,^22^ carbon nanotubes,^23^ and gallium arsenide (GaAs),^24^ into transistor structures. However, current commercial transistors and the most advanced research examples are being manufactured using silicon as semiconducting channel material,^25^, ^26^, ^27^ while the technologies and knowledge in relation to the other materials are still in their infancy in comparison, not being ready yet to fully replace Si. In addition, new‐generation potentiometric chemical sensors with integrated SiNWs offer the promise for the future, taking into account the stability of Si to a wide range of environmental conditions.^28^ The silicon oxide layer (SiO*_x_*) that is typically formed in the ambient conditions is of outmost importance to insulate the semiconductor channel, while enabling the field effect and keeping a good gate potential coupling. In parallel to the silicon oxide, researchers have also been studying the possibility of deposition of high‐*k* dielectric layers preventing the current leakage at ultra‐nanoscales, and improved response in terms of surface potential change,^29^, ^30^ as it will be described later. Finally, Si/SiO*_x_* offers the chemistry possibilities for easy integration of biological receptors and more specific detection. Their potential biocompatibility is a plus for biomedical applications and biological investigations.^31^

In a classical metal–oxide–semiconductor field‐effect transistor (MOSFET), the gate terminal is in direct contact with a thin dielectric layer that surrounds the semiconductor channel. When evolving from the original transistor structure, an ion‐sensitive field‐effect transistor (ISFET) operates in a liquid environment in which the gate electrode is replaced by the charged ionic content of a liquid solution in contact with the dielectric layer, and a relative reference point of a potential difference through the liquid defined by a reference electrode.^32^, ^33^ This structure was proposed for sensing purposes^34^ in the pioneering work by Bergveld in 1970, although with the missing reference electrode. Different chemical reactions occurring at the dielectric–solvent interface depend on the specific composition of the liquid environment and alter the surface charge density and differential capacitance of the electrical double layer (EDL) specifically. In the original configurations, the reference electrode was not used for obtaining the information about the surface charge in the environment, namely sodium‐ion content, with neural activity recording as vision.^34^ Later on, applications of the ISFET sensors were extended beyond recording neural action potential monitoring, and implementing a reference electrode.^35^, ^36^, ^37^ Many of them, including alternative fabrication^38^, ^39^, ^40^ and measuring^37^, ^41^, ^42^ methods, will be reviewed here.

The integration of the nanowires into the sensors made a great impact on ISFET technology. Ion‐sensitive potentiometric devices were revolutionized^43^, ^44^, ^45^ via replacing the planar silicon film (i.e., in metal‐oxide‐semiconductor FET configuration) by its 1D counterpart, initiating a new research field and stimulating further research.^10^, ^26^, ^32^, ^46^, ^47^, ^48^, ^49^, ^50^, ^51^ As a first application,^43^ Lieber's group modified the surface using 3‐aminopropyltriethoxysilane (APTES), obtaining a double surface functionality with the amino groups from APTES and the hydroxyl groups from the silicon dioxide surface. These functional groups can protonate or deprotonate depending on the pH value of the solution, resulting in a linear conductance response of the device in a pH range between 2 and 9. They also functionalized the surface with biotin for specific detection of streptavidin, obtaining detection capabilities down to 10 × 10^−12^ m. Only 3 and 4 years later, they could demonstrate large arrays of individually addressable sensors for multiplexed detection of single viruses^48^ and protein markers at femtomolar concentrations.^45^ Inspired by these works, myriads of publications have exploited the broad range of possibilities in SiNW sensorics (Figure 1), from detection of biomolecules for diagnostic purposes^36^, ^37^, ^52^, ^53^, ^54^, ^55^ to recording of cell activity,^46^, ^56^, ^57^, ^58^, ^59^, ^60^, ^61^ intracellular signal control,^49^, ^62^, ^63^ tissue monitoring,^4^, ^25^ food safety,^64^ and gas^65^, ^66^ and explosive sensing.^67^, ^68^

Definitely, silicon has been attractive also as a photodetecting material due to the convenient integrability with CMOS technology.^20^ The implementation of nanowire growth technology by Lieber,^69^ Yang,^70^ and other groups leads to the explosion of SiNW‐based optoelectronic studies as well. The use of nanowires for optoelectronic applications has several advantages, for instance, i) long absorption distance along the wire, but short carrier transport distance from the NW interface, ii) availability of coaxial heterojunction, iii) strong light trapping by high density of NW arrays, and iv) tunable optical characteristics by controlling the size of the NW.^71^ SiNW photodetectors or photovoltaic (PV) cells have been studied toward the direction to enhance the detection efficiency. To do this, researchers have tried to exploit the optoelectronic properties of organic or inorganic materials as the replacement of the Si‐based photodiode junction structure,^72^, ^73^ or as a functionalizing agent on SiNW FETs motivated by biochemical functionalization in the sensor applications.^32^, ^74^

In the following, we focus on exploring the functional diversity of nanowire‐based devices considering their architecture as well as areas of their applications. To do so, we start from the assumption that a SiNW‐based electronic device, decorated by, e.g., organic complexes (i.e., bio‐ or chemical molecules, polymers, and organic composite materials), is generally a hybrid device. Thus, hybrid devices possess substantially extended functionality, compared to the conventional device, e.g., transistor, resistor, or diodes^35^, ^75^, ^76^ (Figures 1 and 2). Using the aforementioned definition, we can outline several groups of electronic devices that benefit from the available organic coating. The first group is the nanowire‐based biosensors that are able to represent the label‐free assays thanks to the bound biological molecules,^51^, ^54^ pursuing applications such as diagnostics or biomedical research. The second group is represented by the devices for optoelectronics that acquire the higher optical sensitivity or efficiency due to the specific optical properties of the organic complexes adsorbed, for example, photochromic dyes^3^ or photosensitive polymer.^77^ Finally, we name a relatively new group of the nanoscale hybrid devices—the so‐called bioinspired neuromorphic devices^78^ that are aimed to mimic the functions of the biological cells, i.e., neurons and synapses based on the memory property of the nanowire devices using electron trapping or the displacement of ions of the organic layer.

## 2 Fabrication of SiNW Devices

We start with the fabrication methodologies giving as a result a variety of SiNW and multiple electronic device types containing them. Such devices are used for a broad range of applications which are summarized below.

**Figure 3:**
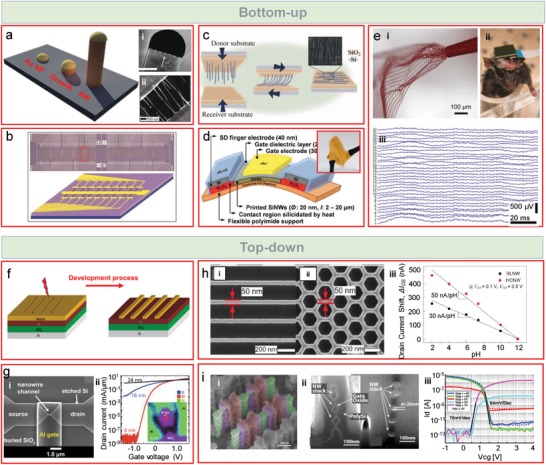
SiNW fabrication approaches and examples: bottom‐up (top panel) and top‐down (bottom panel). a) Schematics of bottom‐up growth of nanowires starting from gold nanoparticle seeds. Right panels show an example of a grown nanowire and its catalyst (upper panel, scale bar 20 nm, adapted with permission.^85^ Copyright 2004, American Chemical Society (ACS) Publications), and two microelectrodes contacted by bottom‐up grown and contact printed parallel nanowires, adapted with permission.^30^ Copyright 2013, Springer. b) An applied example of an array of individually addressable FETs with bottom‐up grown SiNWs. Adapted with permission.^45^ Copyright 2005, Nature Publishing Group. c) Parallel contact printing of SiNWs onto target substrate where the device is going to be fabricated. Adapted with permission.^30^ Copyright 2013, Springer. d) Flexible FET with printed bottom‐up grown SiNWs. Adapted with permission.^92^ Copyright 2016, Wiley‐VCH. e) Syringe injectable mesh nanoelectronics concept, e‐i) with an array of SiNWs FETs for e‐ii,e‐iii) parallel recording of brain activity at 32 separate sites. e‐i) Adapted with permission.^26^ Copyright 2018, ACS Publications. e‐ii,e‐iii) Adapted with permission.^25^ Copyright 2017, ACS Publications. f) Schematics of a typical top‐down fabrication process via EBL. Adapted with permission.^99^ Copyright 2016, PLoS One. g‐i) EBL fabricated SiNW FET. g‐ii) A comparison of various diameters (24, 16, and 8 nm), showing change from 3D to 1D transport. Adapted with permission.^97^ Copyright 2017, Nature Publishing Group. h‐i) Linear and h‐ii) honeycomb nanowires, and h‐iii) pH sensitivity comparison showing sensitivity improvement on the second case. Adapted with permission.^104^ Copyright 2013, IEEE. i) FinFET with i‐i) double‐gate gate‐all‐around, i‐ii) with 20 nm cross section, i‐iii) operating as n‐ or p‐type with the help of polarity control gate electrodes. Adapted with permission.^109^ Copyright 2012, Institute of Electrical and Electronics Engineers (IEEE).

The SiNWs used for most of the aforementioned applications are fabricated following different sets of approaches that one can divide into two categories: bottom‐up and top‐down^79^ processes. During a bottom‐up fabrication, atoms aggregate and assemble forming the desired structures. For the growth of the nanowires, typically, chemical vapor deposition (CVD) of SiH_4_ is done via a vapor–liquid–solid (VLS) mechanism using metallic nucleation catalysts like gold nanoparticles,^80^ whose size can control the diameter of the resulting nanowire (**Figure** **3**a).^81^, ^82^ Modifications on the catalyst shape can also derive on nanowires with cross‐sectional anisotropy,^83^ as was shown for InP, although transferable to other bottom‐up materials like SiNWs. Alternative solutions can also be found in the literature, like solid–liquid–solid (using trisilane, Si_3_H_8_)^28^ or thermal evaporation avoiding the use of contaminant catalysts.^84^ Bottom‐up grown nanowires have desirable advantages, such as the possibility to obtain structures of very small diameter (even down to 3 nm),^85^ and the possibility of in situ doping by incorporation of dopants during growth, and therefore maintaining a high crystallinity.^79^ Heinzig et al.^27^ used such bottom‐up SiNWs to make a remarkable finding, consisting of a reconfigurable FET whose polarity could be tuned without doping steps, opening wide possibilities for logic operations on demand.

For further integration into devices and circuits, the fabricated nanowires need to be transported to the receiver support, where the chip can be finally fabricated with several individually addressable devices (see Figure 3b). Despite the high quality and sensitivity of grown thin nanowires, the resulting device‐to‐device variabilities are high and their performances present low reproducibility and relatively low fabrication yield. In particular, the deposited random SiNW networks can be electrically interconnected following a low thermal annealing process that forms an oxide‐free neck at the junctions between the nanowires.^39^ This allows using a high SiNW concentration to form a homogeneous semiconducting silicon nanonet that improves the quality of the resulting FETs and permits connecting electrodes at larger distances. At the same time, several techniques have been developed to align and orient the SiNWs and obtain a deterministic deposition. Nanowires suspended on a fluid, for example, can be directed through a channel, and the wires will align in the direction of the flow.^86^ If the appropriate microfluidic design is used, with kinked structures in narrow channels, the nanowires can get stuck and trapped at the desired locations for their subsequent contacting between electrodes, forming single nanowire devices, e.g., transistors.^87^ However, these techniques are useful to deposit nanowires selectively and cover small areas on a chip. For deposition of larger areas, a bubble can be extruded at a controlled pressure and expansion rate, from a molten polymer solution where the nanowires are suspended.^88^ SiNWs suffer a shear‐induced alignment along the direction of strain, and the bubble can be ruptured to obtain a flat film with aligned SiNWs. Otherwise, the spreading of the bubble can also be continued onto a flat surface (e.g., a silicon wafer) until the surface is covered by the polymer film. The density of the deposited nanowires can be controlled by tuning the initial concentration or the bubble expansion. Some defects still may appear due to trapped gas during film transfer. Centimeter‐scale arrays are efficiently formed using the Langmuir–Blodgett method,^89^ where nanowires aligned at an air–water interface are patterned on a surface, followed by removal from undesired areas by photolithography prior to the electrode fabrication. Arrays as large as complete wafers can also be transferred and aligned by the contact printing technique, by a directional sliding of the growth wafer onto a receiver substrate.^30^, ^90^, ^91^ During the sliding, the nanowires are detached from the growth wafer and anchored by van der Waals interactions with the receiver substrate (Figure 3c). A lubricant spacer layer can help to prevent the breakage of the nanowires due to friction. The acceptor substrate does not necessarily need to be another wafer, the possibility of printing the nanowires on potentially any surface opens the way to build electronic devices at a low cost on flexible plastic foils (Figure 3d).^92^ Taking the transport onto flexible supports to the limit, this can be done onto a flexible, soft mesh of metallic interconnects, defining tissue‐like structures with SiNW FET arrays, introducing a new concept known as mesh nanoelectronics (Figure 3e), which allows producing cellular‐ or subcellular‐scale sensors on a large area, with mechanical properties similar to tissues, for 3D analysis in living organisms.^26^ The biocompatibility and mechanical deformation capability are very high and enable injection into tissues using a syringe, as it has been demonstrated in mice brains with minimal immune response,^25^ obtaining above 30 simultaneous measuring points in real time (Figure 3e). Generally, the alignment obtained following all these processes is horizontal, but transfer methods to maintain the vertical position of the nanowires have also been described. Horizontal crack layers can be created on the grown nanowires, peeling off the upper layer with tape for the subsequent transport.^93^, ^94^ The vertically grown nanowires can also be encapsulated in an insulating layer of polydimethyl siloxane (PDMS), which will be etched to expose the tips for contacting it with a metal.^95^ However, if the vertical positioning is not needed, the transport step can also be skipped if a local VLS growth is initiated using the resistive heating of a surface, where later a DC electric field can be applied for the alignment of the nanowires.^96^

In spite of all the efforts made, top‐down routes (Figure 3f–i) usually offer a higher yield and device‐to‐device reproducibility, making them more favorable for mass production. These routes involve lithography, etching, and further material deposition steps (Figure 3f), obtaining a high density of devices with precise design and location, crucial for computer technology.^32^ Several lithography techniques exist to define the nanowires: electron beam lithography (EBL),^97^, ^98^, ^99^ focused ion beam (FIB),^100^ nanoimprint lithography,^101^, ^102^ or local oxidation nanolithography using atomic force microscopy.^103^ In some cases, they can even compete with bottom‐up methods obtaining sub 20 nm widths, as exemplified by the 8 nm diameter SiNWs in Figure 3g. Some disadvantages may be encountered, among that we may mention the need for high‐resolution and complex equipment and the use of silicon‐on‐insulator wafers (SOIs) as substrate. Nevertheless, emerging possibilities appear, which cannot be otherwise achieved. Geometries resembling the honeycomb structures are proposed (Figure 3h), which outperform the linear wires occupying the same space, in terms of sensitivity and mechanical robustness.^38^, ^104^, ^105^

The extreme reduction in the semiconducting channel dimensions below 10 nm does not come at any cost. The technology needs to face new drawbacks coming from short channels,^106^, ^107^ namely electrostatic control, source‐to‐drain tunneling, carrier mobility degradation, process variations, and static leakage. The most successful solution to these problems is coming from the construction of FinFETs, FETs with multiple gates or in which the gate surrounds the nanowires for improved control,^108^ as well as vertically stacking them.^109^ De Marchi et al.^108^ combined these approaches by fabricating a double gate, gate‐all‐around FET with vertically stacked SiNWs, where one gate electrode configured device polarity (n‐ or p‐type), while the other one could switch it ON and OFF (Figure 3i). As it was also demonstrated by Seoane et al. using nanoscale InGaAs,^110^ the cross‐sectional shape in FinFETs is a factor to be considered, as triangular cross‐sectional FinFETs have better gate control, higher ON/OFF ratio, and lower OFF current, drain‐induced barrier lowering and subthreshold slope, compared to rectangular or bullet shapes. The cross section would be expected to influence silicon nanowires as well.

The dielectric oxide layer coating the nanowires (gate dielectric) is also an important factor to be considered during fabrication. A common and simple approach is to grow a silicon dioxide layer by thermal oxidation, which shows low interface trap densities.^27^ However, it suffers from ion diffusion, leading to current drift and leakage, if thickness is lower than 20 nm.^111^ This diffusion occurs primarily with alkali cations like sodium and potassium^112^, ^113^ that have a strong presence in biological fluids which are often a target of SiNW FET biosensors. Furthermore, in FETs with extremely reduced dimensions, it can also suffer from electron tunneling leading to current leakage^114^, ^115^ that imposes difficulties gating an FET or retaining the stored charge in the capacitor structure of dynamic random‐access memory (DRAM).^115^ High‐*k* dielectric layers offer a higher capacitance and better gate control, being less susceptible to these effects. Examples like Ta_2_O_5_,^116^, ^117^ TiO_2_,^118^ or SrTIO_3_ 119 are being implemented in nanoscale DRAMs, but are not fully compatible with SiNWs due to reaction either with Si (forming metallic silicides) or with water.^120^ Al_2_O_3_ and HfO_2_ are promising materials that can already be found in several examples of FETs,^29^, ^30^, ^91^, ^111^, ^121^ with an excellent pH sensitivity due to the high density of surface groups that buffer the pH changes of the solution.^122^ pH sensing is a benchmark to assess the quality of ISFETs, and HfO_2_ seems to offer a higher sensitivity, which, unlike SiO_2_, is independent of the ionic concentrations in the solution.^29^ In addition, the chemical inertness in acidic or basic solutions makes it a good candidate for sensing along wide pH ranges.

## 3 Biochemically Configurable Hybrid Devices (Biosensors)

Nanowire‐based potentiometric (bio)sensors represent one of the largest groups in the family of the hybrid devices (see Figure 1). This is due to the enhanced sensitivity of such devices originating from the high surface‐to‐volume ratio of 1D structures. The large surface area of the nanowires is exposed to the surrounding environment, which will lead to the strong influence of the electronic states and thus conductivity of the device. In addition, direct electrical measurements as a label‐free approach are ideal for the design of compact analytical techniques in the biomedical field.

SiNW FETs are sensitive to changes in the ionic composition of the environment, one of the most obvious and recurrent applications is their use as (bio)sensors. The research community in the FETs field has worked for the last years to enhance the sensitivity following different strategies in the design and fabrication of the FETs as well as in the chemical modification of the surface.

### 3.1 Sensing the Ions Using Silicon Nanowires FET (ISFET)

The simplest measurement of ionic composition in a liquid environment can be performed using an FET is pH measurement, due to the high mobility of the protons compared to the other charged species. pH sensitivity is also a parameter used as a benchmark to assess the FET quality. When measuring pH, in an ideal situation and at room temperature, and according to the Nernst equation that governs the processes taking place, the theoretical maximum shift is limited to 59.2 mV/pH.^123^ The electric field that gates the FET come from backgate potential (which will be constant when measuring pH) and the surface potential of the dielectric layer, which will be modulated by the liquid potential and pH, and expressed as

(1) $$V_{\text{surface}}\;\;=\;\;V_{\text{liquid}}-\alpha \cdot 59.2\;\text{mV}\cdot \left(\text{pH‐pI}\right)$$

where *V*_surface_ is the surface potential, *V*_liquid_ is the liquid potential (determined by the reference electrode), α is the relative surface sensitivity with a Nernst limit of α ≤ 1 and 59.5 mV/pH, and pI is the isoelectric point of the surface.^91^ Thus, the surface potential changes will be strongly correlated to the chosen dielectric layer, whose isoelectric point will be different.

Unlike to the theoretical maximum given by Nernst limit, surface imperfections (as well as use of the materials with the lower surface density of OH groups) give, as a result, a usual value of around 30 mV/pH.^7^, ^91^ Distinct strategies attempted to bring the value of this parameter closer to the theoretical limit, such as the patterning of the nanowires in a honeycomb shape,^105^ applying a H_2_ sintering process to increase the number of surface‐binding sites,^124^ the utilization of high‐*k* oxide layers like HfO_2_ and Al_2_O_3_,^111^, ^125^ their substitution for lipid monolayers,^126^ or the immobilization of gold nanoparticles on the nanowire surface to increase the local concentration of hydrogen ions in the vicinity of the nanowire.^53^ While the intrinsic sensitivity limit of the oxide layer cannot be increased, it was demonstrated in recent years that it was possible to go above the Nernst limit by making use of dual‐gated devices to get a capacitive amplification effect.^127^, ^128^

**Figure 4:**
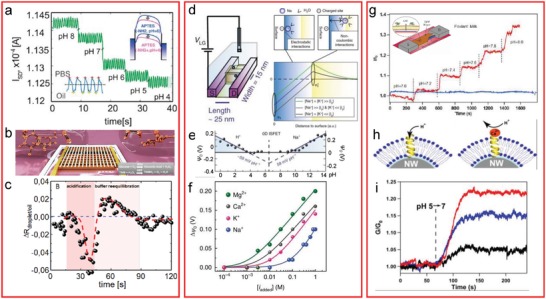
Examples of pH and ion sensing. The left panel shows droplet detection and characterization using SiNW FETs. Adapted with permission.^121^ Copyright 2016, ACS Publications. a) Nanoliter droplet pH calibration, where the APTES‐modified silicon surface gets protonated at acidic pH and deprotonated at high pH (right inset). The droplet is detected as a current peak with a baseline belonging to the continuous oil phase (left inset). b) Schematics of the droplets containing ongoing enzymatic reactions passing over an FET. c) Recording of the glucose oxidation reaction. The middle panel shows selective ion sensing without the use of selective layers. Adapted with permission.^134^ Copyright 2018, Nature Publishing Group. d) Schematics of a 0D ISFET (SiNW with the length of 25 nm and width 15 nm), which contains less than 50 charged sites. On its side, a schematic shows the ion interaction model and the potential as a function of the distance for different ion concentrations. e) The low response of such a short nanowire to pH is shown. f) Calibration of various ions in serum. The right panel shows applications of lipid bilayer–coated FETs. g) comparative measurement of an uncoated (blue) and a coated (red) FET with carbon nanotube porins as proton transporters, after incubation for 60 h in simulated milk. Adapted with permission.^136^ Copyright 2019, ACS Publications. h) Schematics of a nanowire coated with a supported lipid bilayer with an inserted gramicidin channel, through which protons can move unless calcium ions block it. i) pH response of an uncoated FET (red line), a coated FET (blue line), and a coated FET in the presence of calcium ions (black line). Adapted with permission.^139^ Copyright 2009, National Academy of Sciences of the United States of America.

Although a simple pH measurement might not have much interest by itself, there is a number of reasons for which it is interesting to perform it. First, this can be a method to evaluate the quality of the FET. Second, there are countless (bio)chemical and biological reactions that have a pH change as a result, such as microbiological metabolic activity,^129^ DNA amplification by polymerase chain reaction (PCR),^130^ or certain enzymatic reactions, like the case of glucose oxidation by glucose oxidase.^131^ All these reactions can be monitored in real time and label‐free manner with FETs. Schütt et al.^121^ integrated a microfluidic channel design for droplet generation onto a SiNW FET, encapsulating nanoliter sample volumes with enzyme and substrate molecules, allowing us to perform the specific detection of the enzymatic reaction by analyzing the pH (**Figure** **4**a–c). The enzyme catalyzed the oxidation of glucose into gluconolactone, which spontaneously hydrolyzed resulting in liberation of protons, acidifying the medium. Hsu et al.^132^ immobilized the same enzyme on top‐down fabricated nanowires, to obtain a ready‐to‐use device by only incubating the sample without added enzyme. Since the enzymes do not remain bound to the analytes, the device could be reused ten times for the analysis of various samples, with the detection range in the clinical concentrations of interest for glucose. In the case of the detection of DNA amplification products by PCR, the technique consists of the proton release when a nucleotide is incorporated into a DNA strand by a polymerase enzyme. Detection of such a reaction was demonstrated using an electronic packaging containing several SiNW FETs to measure the signal amplification of an immunoassay where the secondary antibody was conjugated with DNA.^133^ However, the immunoassay was taking place in an external location and not on the FET chips.

When the length of SiNWs is shortened to below 25 nm, the density of charged surface sites is extremely reduced, making the FET relatively inert to pH changes.^134^ This characteristic offered an opportunity to study the interaction of other ions neglecting the effect of pH changes or pH‐mediated reactions. Divalent cations could adsorb on the few deprotonated sites of the SiNWs, with a measured effect 230% above the Nernst limit. This increased effect came from the sum of electrostatic interaction of adsorbed cations and non‐Coulombic interactions by the built‐in potential generated by the cations floating at the Stern layer interface. Each cation provides a unique signature to the effect and the technique can be used to make selective blood ionograms without the need for a selective layer (Figure 4d–f).

In contact with complex biological fluids, the biosensor response can be deteriorated through time due to attachment of proteins, cellular debris or cells, namely biofouling.^135^, ^136^ Bovine serum albumin (BSA) and ethanolamine are usual surface‐blocking agents, but the big size of BSA can block the interaction between bioreceptors and target analytes.^137^ A lipid coating of the sensor can protect the surface from fouling, as demonstrated by Chen et al.^136^ using Si nanoribbon FETs. Although a complete lipid coating would isolate it making it insensitive to pH changes, they could introduce carbon nanotube porins as proton channels. These are segments of carbon nanotubes with 10 nm length and 0.8 nm inner diameter that spontaneously insert into the bilayers, and show one order of magnitude higher permeability to protons than to water.^138^ The FETs were still functional and pH responsive after 60 h of incubation in foulants like the bovine serum and milk (Figure 4g). A similar approach was already shown before, by using gramicidin and alamethicin as channels instead of carbon nanotube porins, opening the possibility of including selectivity for certain ions or for new biological studies of transmembrane proteins.^139^ Here, they could show that the FETs could respond to pH by measuring transported protons through gramicidin, unless it was blocked by calcium ions (Figure 4h,i), and the transport through alamethicin when this voltage‐gated channel was activated at +1.5 V. Moreover, the same group showed how the use of light‐gated rhodopsin channels could be used to tune the performance of the electronic device under visible light illumination.^10^

### 3.2 Ion Sensing to Monitor (Bio)Chemical Processes

**Figure 5:**
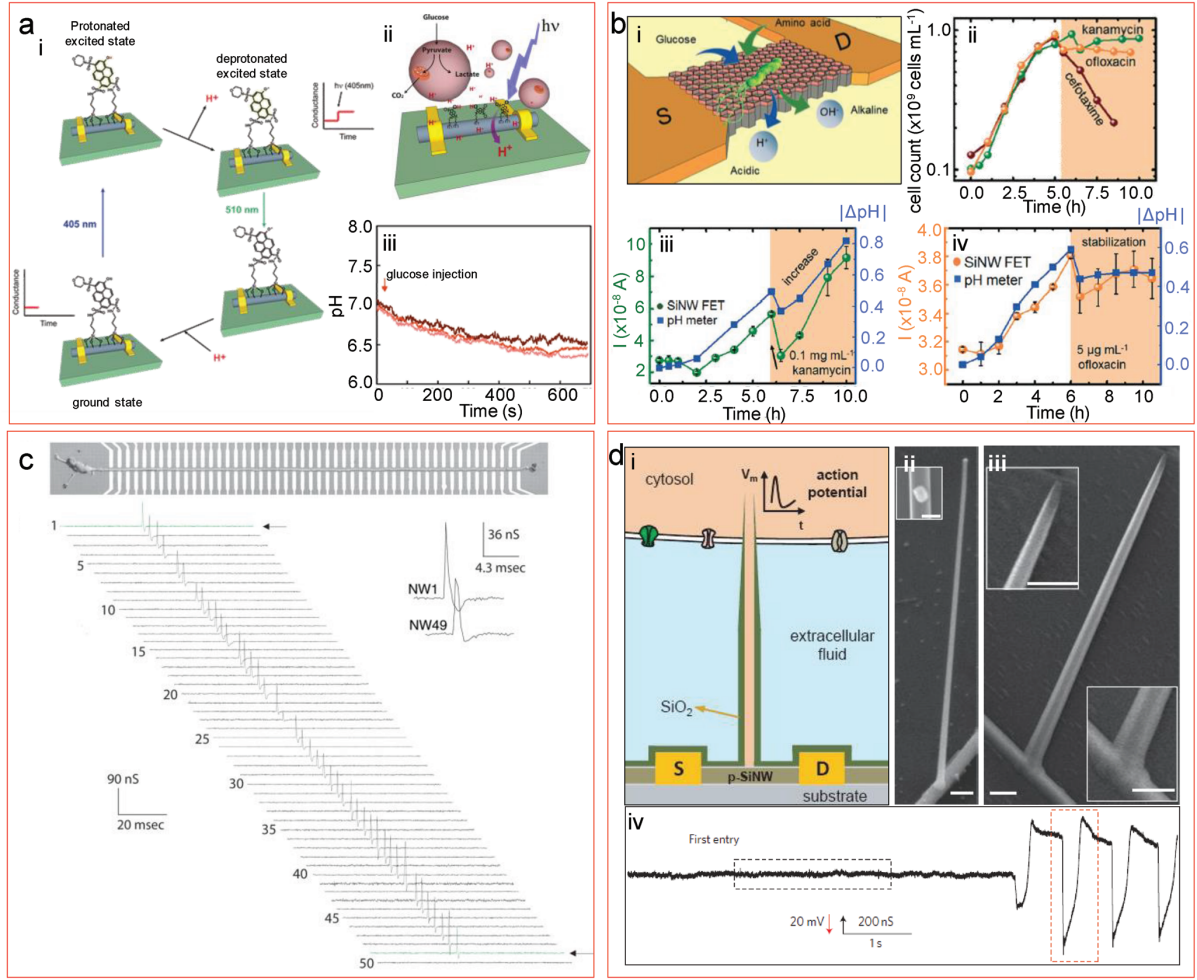
Applications of cell monitoring with SiNW FETs. a) Recording of glucose consumption of Jurkat leukemia cells using a self‐calibrated FET: a‐i) the self‐calibration mechanism, where an 8‐hydroxypyrene‐1,3,6‐trisulfonyl chloride (HPTS)‐modified nanowire is used. HPTS is excited and deprotonated under illumination at 405 nm, causing a conductance change. The deprotonation degree depends on the pH, and the ratio of the current before and after illumination is considered for the measurement. a‐ii) The concept figure where the cells consume glucose producing acidification; and a‐iii) the results are shown. Adapted with permission.^61^ Copyright 2013, ACS Publications. b) Antibiotic screening with *Escherichia coli* bacteria. Adapted with permission.^56^ Copyright 2017, Royal Society of Chemistry (RSC). b‐i) The concept image, with bacteria producing acidification or alkalization depending on the consumed carbon source. b‐ii) Optical effects of kanamycin (bacteriostatic) and ofloxacin (bactericide) cannot be distinguished. b‐ii,b‐iii) This is achieved with FET measurements, where the continuation of the metabolism after kanamycin injection can be observed, while it is completely stopped with ofloxacin. c) Action potential propagation through a neuron axon aligned along 50 FETs. Adapted with permission.^46^ Copyright 2006, American Association for the Advancement of Science (AAAS). d) Intracellular action potential recording by silicon nanotube probes grown from SiNW FETs. Adapted with permission.^49^ Copyright 2011, Nature Publishing Group. d‐i) Conceptual image; d‐ii) germanium nanowire grown from SiNW; d‐iii) final silicon nanotube after SiO_2_ deposition on the germanium wire; d‐iv) conductance trace over time, reflecting the transition from extracellular to the intracellular recording when the nanotube enters the cell.

Among diverse biological or chemical processes, there is a big class that is accompanied with the strong change of ionic composition of the solution, or pH change; for instance, it is an important characteristics of the cellular or bacterial metabolism. Different metabolic reactions result in the production of charged species such as ammonia,^140^ protons,^141^ and organic acid molecules.^142^ Monitoring these processes has important implications in microbiological or cancer research.^142^, ^143^, ^144^ Peretz‐Soroka et al.^61^ measured the acidification caused by glucose consumption and proton production by Jurkat leukemia cells using SiNW FETs with a surface modification that prevented the need of any separate calibration for each FET (**Figure** **5**a). 8‐hydroxypyrene‐1,3,6‐trisulfonyl chloride (HPTS) was immobilized on the SiNW surface, which can be excited and deprotonated upon illumination at 405 nm. This process resulted in a current change, which was different depending on the pH and, therefore, on the protonation state of the molecule. Taking into account the ratio between the excited and the ground state, which was the same for all the different FETs used, the pH value could be deduced without calibration for each of them. These measurements were possible in high ionic concentration (150 × 10^−3^ m), since the protonation/deprotonation processes occurred in the HPTS below the 0.7 nm Debye length. Ibarlucea et al.^56^ demonstrated also the feasibility of measuring the pH changes in high ionic strength using *Escherichia coli* bacteria as model organism growing in microbiologically relevant media (Figure 5b). When the bacteria were grown in M9 minimal salts with glucose as the carbon source, they produced protons, causing the acidification, and when they were grown in Luria Bertani (LB), two phases could be distinguished: the initial acidification via consumption of carbohydrates and further alkalization via amino acid consumption. The work also demonstrates that the activity of bacteriostatic and nonlytic bactericide antibiotics in cell population can be distinguished. In particular, the bacteriostatic drugs, e.g., kanamycin promotes mistranslated protein production but keeps the cells metabolically active. Thus, they still consume glucose that results in the release of the protons, which was detected using the FET devices.

As FETs are intrinsically electrical sensors, they are ideal candidates to measure the behavior of electroactive cells, such as neurons and cardiac cells. Neurons have been placed on top of the FETs, forming nanowire–neuron junction arrays that can be used for detection, stimulation, and/or inhibition of neuronal signal propagation.^46^ By aligning a long axon through several SiNW devices, the propagation of the action potential through the axon could also be traced in real time (Figure 5c). If cardiac cells are placed instead,^58^ their action potential can also be monitored, distinguishing signal kinetics that is associated with inward and outward currents of sodium, calcium, and potassium ions through their respective channels. The intracellular signal is also possible to be measured with the appropriate nanowire architecture. An acute‐angle kinked SiNW was fabricated by Tian et al.,^40^ allowing us to generate a 3D probe that was further modified with phospholipid bilayers to enter single cardiomyocyte cells and measure their interior potentials. This structure facilitates approaching the cells rather than forcing the cells to conform to the substrate. They could find the characteristic phases of cardiac intracellular potentials (resting state, depolarization, plateau, rapid repolarization, and hyperpolarization) although this kinked structure limits the possibilities of size and multiplexing. The same group introduced a variation by growing germanium nanowires from the SiNWs and coating them with SiO_2_, forming silicon nanotubes onto the FETs (Figure 5d), which could also enter the cells via lipid fusion in the same way as the kinked structure.^49^ This structure allowed us to create parallel FETs to record the activity of a single cell or a network of them.

### 3.3 Sensing Biomolecules: Toward Miniaturized Point‐of‐Care Diagnostics

**Figure 6:**
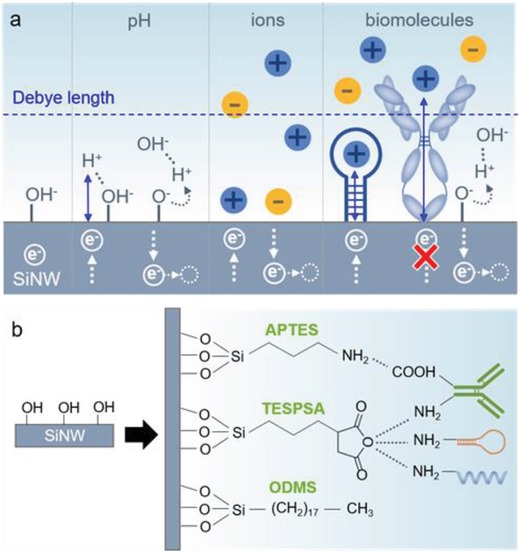
a) Importance of specificity and Debye layer. Only charges under the Debye length limit will have influence on FET conductivity. When there are no receptors (left area), pH and the presence of charged molecules (either specific or unspecific) will determine charge carrier movement. In the presence of bioreceptors, only specific targets will bind, avoiding interference on unspecific charges. However, using large receptors, the “caught” charges will remain above the Debye length, showing no conductivity modulation. pH still interacts, though. b) Examples of SiNW surface modification using silanes containing different functional groups where bioreceptors (antibodies or amino‐modified aptamers or single‐stranded DNA molecules) can be immobilized. Inert silanes can also be attached for chemical passivation.

Miniaturizing, parallelizing, and simplifying the detection techniques is of utmost importance to create the necessary tools that will increase the throughput of research and clinical analysis in medical centers. Point‐of‐care diagnostic sensors allow performing the measurement next to the patient,^145^ enabling quick decision taking even in remote places far from the hospitals or laboratories where the traditional analyses usually take place. Such sensors need to be highly sensitive, rapid, and specific toward the analyte of interest. During a simple pH measurement with a bare FET, the selectivity is uncontrollable. Total change of ionic content contributes to the signal modulation (**Figure** **6**a). According to the classical biosensor definition,^146^ a bioreceptor is needed, together with the transducer, to convert a physical interaction with the analyte of interest into an electronic readable signal. In this way, the device will respond solely and specifically to the desired analyte. Particularly in FETs, the response depends on the charge sign of the analyte molecule and the transistor type (n or p). Molecules with a net positive charge will have an electrostatic attraction effect on negative charge carriers (electrons), or repulsive effect on positive carriers (holes), modulating the conductivity. The opposite will happen with molecules presenting a net negative charge. This will be observed as a shift in the transfer characteristics (gate voltage vs current values), and therefore a measurable shift in the threshold voltage. The shift will depend not only on the net charge, but also on the amount of molecules caught by the bioreceptors, which is useful to deduce the concentration of these molecules in the sample with the help of a calibration curve. However, one of the serious issues for the silicon nanowire sensors is represented by complexity and molecular heterogeneity of the biological samples that are typically analyzed. Biodetection is possible within the distance of the Debye length, which is comparable to the radial dimensions of the SiNWs but dependent on the ionic strength of the sample.^14^, ^37^ This distance becomes shorter with the increase in the ionic strength. Biological fluids as well as typical laboratory buffers simulating physiological conditions (such as phosphate buffered saline (PBS)) have a high ionic strength of 162.7 × 10^−3^ m. The Debye length at this level is only 0.7 nm. Anything above it will remain undetectable (Figure 6). Therefore, the buffer needs to be significantly diluted to increase the thickness of the Debye layer or use small receptors and short linker molecules during functionalization as alternative.

To make a sensor specific, bioreceptor molecules need to be immobilized directly at the surface of the electronic channel, as mentioned. Molecules can be directly adsorbed on the SiNW surface through hydrogen bonding, van der Waals forces, or hydrophobic interactions, but this might not be the optimal strategy, since the long‐term stability will be compromised.^147^ Changes in the environment properties such as pH, ionic strength, temperature, or polarity of the solvent could reverse the binding process. Chemical modifications of the silicon surface are usually done to create strong bonds between the bioreceptor and the nanowire. The chemistry of silicon is well known and, unlike other materials also implemented in FETs like carbon nanotubes,^148^ graphene,^149^ or MoS_2_,^150^ it allows easy implementation of covalent bonds on its hydroxyl‐rich surface. Silanization is a classical silicon surface modification technique extensively used on silicon nanowires,^65^, ^151^, ^152^, ^153^, ^154^ where silicon atom containing molecules covalently bind leaving a variety of functional groups, depending on the specific chosen silane molecule. Some examples include APTES with amino groups,^55^ 3‐(triethoxysilyl)propylsuccinic anhydride (TESPSA) with succinic anhydride functionality,^155^ or octadecyldimethylmethoxysilane (ODMS) with no reactive groups for passivation^35^ (Figure 6b). Silanes with reacting functional groups can be used as short cross‐linkers for bioreceptor immobilization, and inert ones for silicon surface passivation.^155^ When bottom‐up SiNWs are used, these can be processed in solution to avoid functionalizing also the silicon wafer where they will be printed.^156^

**Figure 7:**
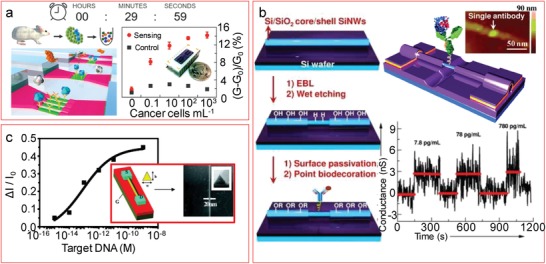
Biosensing with classic bioreceptors (antibody, DNA). a) Rapid cancer cell detection down to 0.1 cells mL^−1^. Adapted with permission.^157^ Copyright 2016, ACS Publications, https://pubs.acs.org/doi/full/10.1021/acsnano.5b07136, further permissions related to this figure should be directed to ACS. b) Single Influenza antigen detection with SiNW FETs modified with single antibodies. Adapted with permission.^158^ Copyright 2014, Wiley‐VCH. The real‐time detection shows three identical peaks independent of the concentration incubated, showing that only one antigen was binding each time. c) DNA detection down to 1 × 10^−15^ m using SiNWs with triangular cross section. Adapted with permission.^159^ Copyright 2011, ACS Publications.

The classic bioreceptors are antibodies and DNA that will bind to antigens with a net charge or hybridize to complementary DNA (with a negatively charged backbone), respectively, modulating the FET conductivity. Kim et al.^36^ reached a 5 pg mL^−1^ detection limit for cardiac troponin I (cTnI) utilizing honeycomb shape SiNW‐FETs patterned by electron beam lithography with immobilized monoclonal antibodies, one order of magnitude below the suggested threshold limit. Tran et al.^157^ developed an intraoperative biosensing platform with electron beam lithography defined nanowires and immobilized anti‐cytokeratin antibodies to detect disseminated tumor cells in lymph nodes and circulating tumor cells in peripheral blood within an hour, demonstrating that the detection could be done within the surgery time frame to avoid unnecessary secondary surgeries to patients (**Figure** **7**a). Wang et al.^158^ formed a hydrogen‐terminated silicon trench in the nanoscale by HF/NH_4_F wet etching, with the size comparable to that of an antibody. They used this trench to immobilize by photochemical hydrosilylation of an antibody against the H1N1 Avian Influenza Virus (AIV), while passivating the rest of the surface with the nonreacting octadecyltrichlorosilane (OTS). By integration with a microfluidic channel, they could demonstrate the binding of single H1N1 Hemagglutinin antigens (Figure 7b). Regarding SiNW FETs with DNA as bioreceptors, Karnaushenko et al.^92^ developed a flexible biosensor for H1N1 AIV DNA with bottom‐up grown nanowires deposited in a parallel array by contact printing. By immobilizing an amino‐modified single‐stranded DNA (ssDNA) receptor on the carboxylated surface, the hybridization of the target DNA strands at picomolar levels could be detected as a conductivity increase due to the accumulation of negative charges of the DNA molecules. Gao et al.^159^ fabricated 20 nm narrow nanowires by anisotropic wet etching and, after a further amino modification of the surface, they immobilized carboxy‐terminated ssDNA, allowing us to perform the detection of H1N1 DNA down to 1 × 10^−15^ m (Figure 7c).

The downside of using antibodies or large DNA sequences is their size as mentioned in Figure 6. For the aforementioned classic bioreceptors, and following standard functionalization protocols, measuring real clinical samples like blood or serum would not be possible, being necessary to process the sample by desalting or dilution, which can be more challenging when the detection needs to be done at patient's site. Diluting the sample to increase the Debye length above the size of the receptor to allow target binding detection might be possible, but the correct function of the bioreceptors is compromised. In addition, the pH of the solution might be unstable in diluted buffers, and the addition of charged entities could induce pH changes affecting to the target charge and therefore to the FET signal.^160^ A current hot topic in nanowire‐based biosensors research is precisely to develop methods that allow increasing sensitivity to detect molecules at a physiological level, overcoming the Debye length limitation. These strategies can be related to the use of alternative surface chemistry methodologies, the use of smaller receptors, or the measurement in an unconventional format not usually associated with ion‐sensitive FETs.

### 3.4 Alternative Surface Chemistry

**Figure 8:**
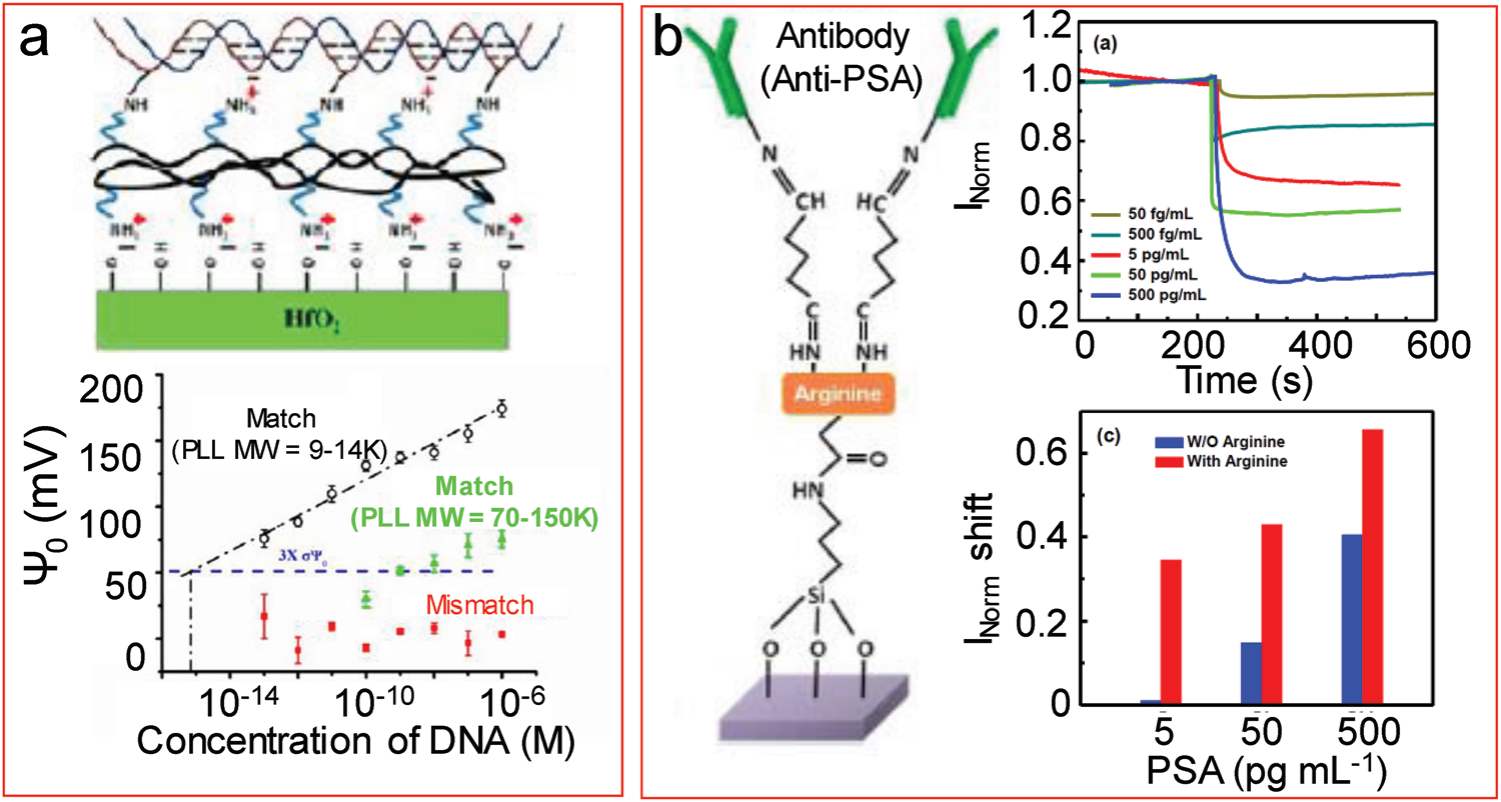
Alternative functionalization strategies for improved biodetection. a) Horizontal immobilization of DNA electrostatically deposited on PLL layers. A large detection range between 100 × 10^−15^ m and 1 × 10^−6^ m was obtained. Adapted with permission.^111^ Copyright 2012, ACS Publications. b) Inner positioning of arginine between glutaraldehyde and APTES to create and electrostatically compressed gap between the receptors and the surface. The detection in real time was possible down to 50 fg mL^−1^ and the comparison showed clear improvement compared to absence of arginine. Adapted with permission.^161^ Copyright 2012, ESG.

The most frequent DNA immobilization strategies involve the use of an ssDNA with a modified end to anchor it to the surface. If the strand is standing up, the charges of the opposite end will be far from the surface, poorly contributing to the signal. A solution to this problem is the horizontal positioning of the strands, which would still allow the hybridization with the complementary strand. Dorvel et al.^111^ electrostatically immobilized a poly‐l‐lysine (PLL) layer over the negatively charged surface of the high‐*k* hafnium oxide used as the top oxide layer (**Figure** **8**a). This created a positive layer of polymer onto which the ssDNA could be horizontally attached due to its characteristic negatively charged backbone. All charges of the ssDNA as well as the ones of the target strand after the hybridization were closer to the surface, helping to obtain an improved signal in femtomolar levels. Here it is important to consider the molecular weight (MW) of the polymer. Due to roughness issues, small molecules (MW = 9–14k) were shown to give better results compared to larger ones (MW = 70–150k).

This technique of utilizing layers of opposing charge also helps to electrostatically attract the analyte and to compress the gap between the analyte and the surface, resulting in a larger amount of detectable signal inside the Debye length. Wu et al.^161^ applied a similar approach with antibodies as bioreceptors instead of DNA. They introduced a variation to the widely used modification protocol based on APTES followed by glutaraldehyde^162^, ^163^, ^164^, ^165^ for antibody immobilization on silicon. They placed an intermediate molecule, an arginine linker, between APTES and glutaraldehyde on nanobelt FETs (Figure 8b). This showed several advantages: it is a short length molecule, but creates a layer that helps avoiding direct contact between the antibody and the surface to maintain intact the function of the recognition site of the antibody. In addition, there was an attractive force between the antigen (prostate specific antigen, PSA) and arginine, which helped to compress the gap between the semiconducting channel and the antigen, improving the signal compared to the detection without arginine. The lowest readable signal was decreased from 5 pg mL^−1^ PSA concentration without arginine to 50 fg mL^−1^ with arginine linker.

Sometimes, during the biofunctionalization process, the surface functionality needs to be changed or activated. This can be achieved without significantly increasing the distance from the surface, using the so‐called zero‐length crosslinkers. For example, an amino‐surface can be further modified with succinic anhydride,^92^, ^166^ whose ring structure will spontaneously open in the aqueous environment resulting in a carboxylated surface. Further, a carboxylated surface can be activated using carbodiimide chemistry in order to bind amino‐containing molecules.^167^

**Figure 9:**
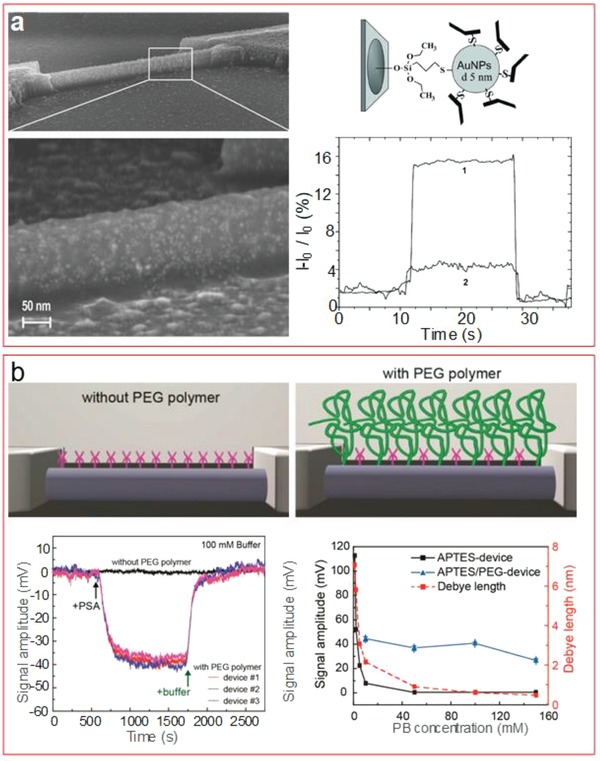
a) Scanning electron microscopy image and sketch of SiNW modification with antibody fragment decorated gold nanoparticles. The real‐time measurement demonstrates the improved response of the modification, showing stronger signal change. Adapted with permission.^53^ Copyright 2017, Elsevier. b) Surface co‐immobilization of APTES and PEG, improving the signal as shown in the real‐time measurement graph compared to the surface with only APTES. This strategy allows measuring in high ionic strength, as shown in the right graph with measurements up to 150 × 10^−3^ m. Adapted with permission.^11^ Copyright 2015, ACS Publications.

As a signal amplification technique, bioreceptors can be attached through gold nanoparticles instead of a direct immobilization to the linkers. Presnova et al.^53^ compared the signal obtained using antibody fragments, cut by 2′mercaptoethylamine (2‐MEA), on 5 nm gold nanoparticles attached to the nanowire surface via silane chemistry, to the one of whole antibodies without nanoparticles (**Figure** **9**a). The sensitivity obtained was fourfold higher, with a detection range for PSA between 23 fg mL^−1^ and 500 ng mL^−1^, and successfully detecting it in serum samples. The authors suggest that the antibody fragments present their active sites oriented toward the nanoparticles, which amplify the electric field near the nanowire surface.

A more general methodology was described by Gao et al. to efficiently increase the Debye length, useful to perform detection in high ionic strength solutions regardless of the bioreceptor used. It involved the modification of the surface with a porous and biomolecule permeable polymer (Figure 9b). More specifically, they introduced a mixture of APTES and polyethylene glycol (PEG)—modified silane. PEG changed the dielectric properties in aqueous solutions, increasing the Debye length, while APTES would allow a further modification with the required bioreceptor to provide the FET with specificity. 100 × 10^−6^ m PSA could be detected in real time in a 150 × 10^−3^ m phosphate buffered (PB) solution, while the buffer had to be more than ten times diluted in order to observe a signal change when no PEG was present. Then, at 100 × 10^−3^ m PB, a clear sensing response was observed between 10 × 10^−6^ and 1000 × 10^−6^ m. Although the detection was not specific due to absence of bioreceptor, this was later accomplished^168^ using an aptamer immobilized on a graphene FET and showing a biosensing response down to 1 × 10^−6^ m, with regeneration possibility by a separation of the analyte using guanidinium chloride.

### 3.5 Small Receptors

**Figure 10:**
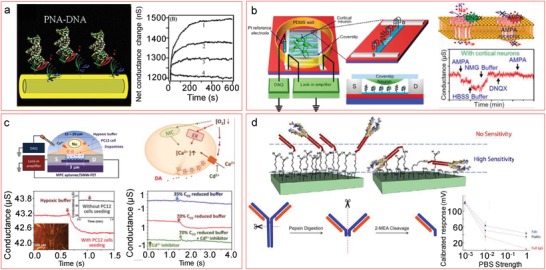
Alternative, smaller bioreceptors. a) DNA detection using PNA, including real‐time measurements down to 10 × 10^−15^ m (curve 4). Adapted with permission.^44^ Copyright 2004, ACS Publications. b) Use of aptamers to detect K^+^ efflux from cells. Graph shows the real‐time measurement where stimulation with α‐amino‐3‐hydroxy‐5‐methyl‐4‐isoxazolepropionic acid (AMPA) is followed by K^+^ efflux, while 6,7‐dinitroquinoxaline‐2,3‐dione (DNQX) blocking brings the absence of response upon repeated AMPA stimulation. Adapted with permission.^57^ Copyright 2017, ACS Publications. c) Dopamine detection on pc12 cells under hypoxic stimulation. The right sketch shows the proposed mechanism, by which hypoxia stimulates Ca^2+^ uptake, which further triggers dopamine release. The real‐time measurements show signal change when hypoxia is generated, and a comparison for different oxygen concentrations, including absence of signal change when Ca^2+^ channels are blocked with cadmium ions. Adapted with permission.^171^ Copyright 2013, ACS Publications. d) Measurement improvement using antibody fragments and low receptor density. The increase in the mobility of the receptor due to low density improves the probability of falling inside the Debye limit. When the receptor is small (antibody fragment compared to full antibody), the signal is also improved. Antibodies can be cut by pepsin digestion and cleavage with 2‐MEA, allowing measurements in undiluted PBS, as shown in the graph. Adapted with permission.^175^ Copyright 2012, ACS Publications.

An alternative to the classic receptors like DNA or antibodies is the use of smaller receptors (**Figure** **10**) to help maintaining the target molecules inside the Debye length. Aptamers, for example, are small molecules with either nucleic acid or peptide structure that bind specific ligands, created by an in vitro affinity selection among a large amount of random sequences.^169^ Although there is a limitation on the available target molecules that can be detected using aptamers compared to antibodies, the research during the last years has shown that the ongoing research is rapidly widening it.^170^ Due to their size advantage, there is an increasing interest on them in the FET community, as demonstrated by the large amount of ultrasensitive silicon nanowire‐based aptasensors developed during the last years for detection of neuronal potassium ion efflux^57^ (Figure 10b), dopamine^171^ (Figure 10c), thrombin,^52^ PSA,^172^ and antivascular endothelial growth factor (VEGF)^173^ among others.

For the specific case of DNA detection, there is an added disadvantage coming from the traditional use of ssDNA as bioreceptor. It consists on the repulsive forces due to the negative charges in the phosphate groups of the molecule backbone. Usually high ionic buffers or divalent cations such as Mg^2+^ or Na^2+^ are used to screen those charges and improve the hybridization yield, which would be counterproductive with an FET measurement. The ssDNA can be substituted for a peptide nucleic acid (PNA),^174^ a synthetic polymer with single base specificity that binds to the DNA with greater affinity than the complementary DNA strand itself (Figure 10a). The PNA is not charged, meaning the absence of such repulsive forces and thus improving the binding. Hahm and Lieber^44^ made a demonstration by immobilizing biotinylated PNA on an avidin functionalized nanowire surface. They could perform a real‐time DNA detection with the limit at 10 × 10^−15^ m to distinguish the ΔF508 mutation site in the cystic fibrosis transmembrane receptor (CFTR) gene from the wild type.

Instead, if the receptor is an antibody, it can be fragmented into smaller parts. Elnathan et al.^175^ compared the signal obtained by using full antibodies with different size reductions and fragments (Figure 10d). On one hand, they cut via pepsin digestion the Fc portion, which is useless during the biosensing. On the other hand, they further reduced the size by separating the two Fab regions through a cleavage of the disulfide bonds using 2‐MEA. Antibodies against cardiac troponin were chosen for the test. They first showed that the smaller the fragment, the improved the response toward a single 40 × 10^−6^ m concentration of cardiac troponin T (cTnT) in every ionic strength that they compared, up to 1× PBS (around 150 × 10^−3^ m ionic strength). Then, by using the F(ab′)2 fragment (both recognition sites bound with the Fc portion digested), they could demonstrate real‐time picomolar–nanomolar detection in undiluted desalted serum. The influence of the receptor density was also analyzed by comparing different incubation times for the used linker, glutaraldehyde. A moderate value was preferable, allowing us to have an appropriate number of receptors with enough flexibility, to adopt conformations closer to the nanowire surface.

Other lesser‐known small molecules can present a good affinity toward some concrete analytes. It has been possible to exploit the known intermolecular interactions to apply them in the detection of prion proteins using thiamine,^176^ Influenza A virus Hemagglutinin Human H1 and Avian H5 using glycans^177^ or histidine‐tagged proteins with Ni^2+^.^156^ Detection capabilities using different receptors are shown in **Table** **1**.

**Table 1:** Detection capabilities demonstrated for various receptors

Bioreceptor	Detection
Antibody	6 × 10^−15^ m (Ebola VP40 protein),^55^ single virus (Influenza A),^48^ single proteins (Influenza A Virus Hemagglutinin)^158^
Aptamer	10 × 10^−12^ m (dopamine),^171^ 200 × 10^−12^ m (α‐thrombin)^37^
DNA	1 × 10^−15^ m (25‐mer DNA),^159^ 40 × 10^−12^ m (18‐mer DNA)^92^
PNA	10 × 10^−15^ m (31‐mer DNA)^44^
γ‐pyrone	50 × 10^−15^ m Fe^3+^ 126
Thiamine	40 × 10^−12^ m (human prion protein PrP^c^)^176^
Glycan	50 × 10^−18^ m (Influenza A Virus Hemagglutinin)^177^
Ni^2+^ quelated on AB‐NTA	Single His‐tag F1‐ATPase motor protein^156^

AB‐NTA: Nα,Nα‐bis(carboxymethyl)‐l‐lysine.

### 3.6 Alternative Measurement Modes

**Figure 11:**
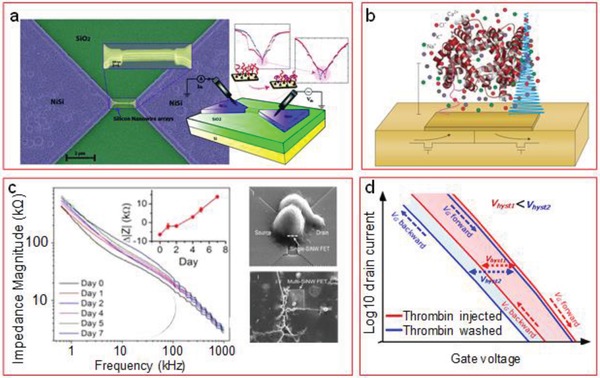
Alternative measurement modes for FET biosensors. a) Stacked SiNWs for measuring the hysteretic behavior on the memristive properties during biomolecule binding. Adapted with permission.^288^ Copyright 2017, RSC. b) Conceptual image representing the use of high‐frequency impedance signals, avoiding electrical double layer formation. Adapted with permission.^184^ Copyright 2015, Nature Publishing Group. c) Application of the impedimetric method to monitor cell growth and differentiation. Adapted with permission.^5^ Copyright 2015, ACS Publications, https://pubs.acs.org/doi/10.1021/acsami.5b01878, further permissions related to this figure should be directed to ACS. d) Conceptual graph showing hysteresis variation in transfer curve of a SiNW FET according to variations in analyte (thrombin) presence. Adapted with permission.^37^ Copyright 2018, Multidisciplinary Digital Publishing Institute (MDPI).

Having mentioned that the reason of sensitivity loss in high ionic strength samples comes from the screening of the signal by excess of nontarget ions in the solution, it seems an obvious strategy trying to get rid of those ions. The common procedure for this is to dilute the sample. If one carries this to the limit, the way to get rid of all ions is simply removing the liquid from the nanowire surface. Some works have already demonstrated that it is possible to measure highly sensitive detection of charged molecules in air. Choi et al.^178^ compared the transfer characteristics of silicon nanowire FETs in both dry and wet environments after sequential addition of molecule layers with opposite charge: APTES, polysodium–styrenesulfonate (PSS), and polyallylamine hydrochloride (PAH). Their results showed that the sensitivity in dry conditions was significantly higher, although the results were also more irregular and unstable due to the nonuniformity of the layers in such conditions. Puppo et al.^179^ also demonstrated that the biosensing in air was highly sensitive, obtaining femtomolar detection limits for VEGF for complex human breast tumor extracts. Furthermore, they also demonstrated that it was possible to determine the pH of the sample in which an FET had been previously immersed due to the formation of a wet film at the nanowire surface, where protons move freely modulating the conductivity of the semiconductor channel.^180^ However, they pointed out that the aforementioned instability could be related to humidity variations in the environment.^181^ Water molecules can adsorb to the surface layer that might alter the results. Therefore, dry measurements must be done under an environment with controlled humidity. A particularly unusual measuring mode was used in the last case, based on the memristive properties of the device (see the scheme in **Figure** **11**a). This new biosensor type was demonstrated by Carrara's group,^42^ showing that the binding of charged biomolecules opened a gap between the minimum current peaks in the semilogarithmic output curves. The gap was associated with the larger energy that was necessary to transport the charge carriers in comparison to the bare device, and its width depended on the amount of molecules bound and their charge sign. The technique showed very high sensitivity, as demonstrated by detection of PSA in atto‐ and femtomolar concentrations, by performing the measurements in dry conditions after incubation and cleaning.^172^ However, measuring in dry can be irregular, unstable, and with higher variations than measuring directly in a liquid sample using controlled potentials via reference electrode.^178^ Ibarlucea et al.^55^ demonstrated that the technique can be used to measure in liquid. Moreover, the initial width of the gap can be controlled and set on demand making use of the gate electrode, allowing us to measure the target molecules with the charge sign that close the gap further, and which would not be possible to measure when the initial condition of the device is to show the gap already closed.

In the works published by Carrara's group, the binding of molecules at such low concentrations was also confirmed by impedance measurements under alternating current (AC) excitation, which demonstrates that impedance measurements using FETs are valid for ultrasensitive detection.^172^ The use of AC excitation to measure beyond the Debye length with FETs was first proposed by Kulkarni and Zhong using carbon nanotube FETs.^182^ They suggested that at high frequencies the ions do not have enough time to form the EDL, and the fluctuating dipoles of the analyte modulate the surface potential of the nanotube. Biotin binding on a streptavidin layer above the Debye length in 100 × 10^−3^ m ionic strength buffer could be sensed. Later, Laborde et al.^183^ proved it as a feasible technique with CMOS chips, imaging the attachment and movement of cells in real time in growth medium. As pointed out by Ingebrandt,^184^ the use of this technique with nanoscale sensors might be of a significant impact for biosensing (Figure 11b). Impedance measurements have already been used with a multiplexed system containing 16 SiNW FETs, to detect DNA hybridization.^41^ The measurements were done at 100 kHz, where the frequency‐dependent transconductance was at its maximum. Interestingly, in this work, the authors observed that the shift in the impedance signal was opposite to that expected when the ionic strength of the sample was high, and no explanation could be found. Impedance technique was also used for cell analysis by Lin et al.^5^ (Figure 11c). They could observe neuron adhesion, growth, and differentiation through several culturing days by measuring the impedance changes on the nanowire device. The cell attachment was done on a poly‐d‐lysine layer covalently attached to the silanized surface. It is well known that cells attach and flatten on surface coated with the polymer due to the difference between the polycationic polymer and the polyanionic cell membrane.^185^, ^186^ This property was exploited to electrostatically attract the cell membrane closer to the surface.

Although without particular improvement toward the measurement in high ionic strength, a new biomolecule quantification method was recently demonstrated based on the hysteretic behavior of the transfer curves^37^ (Figure 11c). The presence of polarizable molecules provokes a change in the hysteresis between the measured currents in the forward and backward sweeps of the gate voltage. The hysteresis width is related to the concentration of molecules. The demonstration using thrombin as target showed that the measurable level was in the nanomolar range, while the threshold voltage shift method allowed measuring down to picomolar levels, making it possible to extend the detection range. This new method opens a new way to quantify polarizable molecules but which have little or no net charge, and that could not be measured with the traditional threshold voltage shift method. It was also recently shown, however, that neutral molecules can also be detected by measuring in the linear regime of the FET.^187^ Despite these molecules do not have a direct electrostatic effect on the charge carriers of the nanowires, they contribute with a dielectric constant increase at the oxide layer, increasing the gate capacitance, and therefore causing a modulation on the effective gate coupling and final FET conductivity.

An overview of the detection capabilities of alternative measuring modes is shown in **Table** **2**.

**Table 2:** Detection capabilities demonstrated for various detection modes

Measurement mode	Detection
Threshold voltage shift	100 × 10^−15^ m (10‐mer DNA, analog of breast cancer miRNA)^111^
Conductance change	1 × 10^−15^ m (25‐mer Influenza DNA)^159^
Memristor (voltage minima gap)	23 × 10^−18^ m (prostate‐specific antigen)^172^
Impedance	33 × 10^−18^ m (prostate‐specific antigen)^172^
Gating hysteresis	20 × 10^−6^ m (α‐thrombin)^37^
Dielectric constant change	20 µg/mL (anti‐Avian Influenza antibody)^187^

### 3.7 Biosensor System Integration

**Figure 12:**
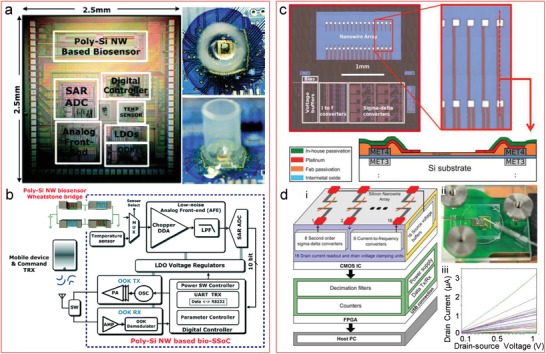
CMOS platforms with integrated SiNW FETs. a) Platform comprising poly‐SiNW FETs and temperature sensors with a multiplexer for biosensing of liquid samples. b) The architecture shows the overall schematics of the platform, where the input can be chosen with the multiplexer, amplified, converted to digital signal, and communicated wirelessly for real‐time temperature monitoring and calibration capability for the FETs. Adapted with permission.^189^ Copyright 2013, RSC. c) CMOS chip with integrated contacts for SiNW FETs, used for biochemical sensing. d‐i) Schematics of the building blocks forming the complete system, including readout electronics, SiNW FET array, and a field‐programmable gate array for data processing and communication to a computer. d‐ii) The picture of the whole packaged system including the polymer fluidic tank for biosensing. d‐iii) Output curves for 27 SiNW FETs. Adapted with permission.^188^ Copyright 2015, ACS Publications.

The rapid demographic changes with increasing growth of the population and limiting resources require improving the biomedical technologies toward cost–effectiveness, compactness and modularity, and higher throughput. The operation of portable and compact devices by ordinary people opens existing possibilities allowing easy, fast, and cost‐efficient implementation of different application‐specific (bio)chemical processes in order to conduct on‐site testing at home, office, or school. Despite some achievements made in recent years, the combination of all these features in a single portable biosensing system proves to be a difficult task. Considering the miniature size and all the previously described fabrication and chemical modification methods, nanoscale ISFETs packaged in a small chip can be ideal candidates for this, integrated with additional circuits and in lab‐on‐a‐chip setups where further processing steps can be automatized. New approaches for connecting nano‐ to microworld involve higher requirements to system integration approaches as fabrication of dense, reliable, and scalable sensor arrays enabling massive parallelism and high throughput, including readout and front‐end electronic systems. The key features of a modular sensing platform should include compactness of conditional electronics, disposable, and cost‐effective sensor part, user‐friendly software interface, integrated packaging solutions for the measurement in liquid medium, and multiplexing capabilities. The CMOS technology fulfills all these tasks and brings many advantages in integrated chips for biochemical applications (**Figure** **12**).^188^, ^189^ CMOS‐based biosensors make possible to have sensor chip and readout part physically on the same substrate; sensor signal digitalization is directly after measurements; and parallelization of readout and compensation of sensors can be implemented. Monolithic integration of other processes such as signal amplification and wireless communication is also enabled decreasing the cost and size, and increasing the mobility of the overall device. An additional advantage of the integration is that it allows making use of several transistors simultaneously without using large circuitry, correlating the results of identical sensors, or applying different ones for various parallel sensing applications. This capability is essential for solving important tasks relevant for, e.g., large‐scale biological screening, highly multiplexed assays, and clinical diagnostics.

Livi et al. presented a sensor system consisting of top‐down fabricated SiNW FET arrays and CMOS readout.^190^ The CMOS circuitry comprised a sigma–delta modulator and a current‐to‐frequency converter connected to individual nanowires allowing us to measure up to 16 simultaneous sensors in real time. Later this group demonstrated^188^ a monolithic integration of an array of FETs onto a CMOS chip with the readout circuit on the same substrate and conducted cardiac troponin T for ten nanowires. Huang et al.^189^ presented a fully integrated polysilicon nanowire‐based biosensor with wireless acquisition circuit in CMOS technology. The chip architecture consisted on a low‐noise front end, a 10 bit successive approximation analog‐to‐digital converter, low dropout voltage regulators, a digital controller, a wireless transceiver, and a temperature sensor. They were able to distinguish picomolar levels of N‐terminal prohormone brain natriuretic peptide (NT‐proBNP) among other markers. A portable sensing system for SiNW FET arrays was also presented by Voitsekhivska et al.^191^, ^192^ demonstrating real‐time multiplexing measurements using custom‐designed CMOS‐based analog multiplexer chip integrated on an SD‐card test board adaptor, allowing reliable and stable multiplexed electrical characterization of sensor elements in the liquid medium. The readout circuit architecture behind the multiplexer is based on the transimpedance amplifier with analog‐to‐digital converter, allowing digitizing obtained current with low noise and wide bandwidth. Real‐time measurements could be performed with up to 32 FETs in an array or 240 FETs in matrix configuration.

Additional nonelectronic solutions can be added to small‐sized chips, in the form of microfluidic channels, that can manipulate the sample or other reagents. These include from simple channels for delivery and separation of low‐volume samples^193^ to more complex structures such as mixers,^194^ bioreactors,^195^ concentration gradient generators,^196^ nanoliter compartments,^197^, ^198^ and pumps for fluid movement.^199^ The downside of integrating microfluidic structures with FETs is that the streaming potential can introduce an additional noise in the measured signal, especially when low ionic strength is used. An electric field is built along the stream, which brings a gradient in the surface potential along the flow direction. Fluctuations in flow velocity will modify the surface potential inducing the noise.^200^, ^201^ A solution to this problem was proposed^202^ by coating the microfluidic channel with a thin silver film, and connecting a reference electrode at the inlet and at the outlet of the channel. The demonstration was done using piezoelectric pumps for the liquid flow, which can present large flow velocity fluctuations.

A complete system comprising the FETs and all the surrounding structures with various functions are ideal to perform complex tasks such as analyzing complex fluids and their dynamics.

## 4 Hybrid Nanowire Devices: A Solution for Optoelectronics

In parallel to the biosensing platforms, SiNWs are attractive building blocks for the integration in optoelectronic devices based on the deep understanding of fabrication processes, as well as in material physics. The structural property of nanowires, e.g., a large aspect ratio, provides the unique advantages to the conventional photonic‐integrated devices, keeping low power consumption. As light‐emitting devices such as light‐emitting diodes (LEDs) or lasers, SiNWs do not represent the best solution, due to their indirect energy bandgap structure, which significantly reduces gain coefficient.^203^ However, SiNWs offer great opportunities and interests for light‐detecting devices, such as photodetectors, photovoltaics, plasmonics, or negative index metamaterial devices, in ultraviolet (UV) to near‐infrared (NIR) region. Si‐based photodetectors can convert optical signal to electrical current or voltage with reasonable efficiency.

In spite of existing various direct bandgap semiconductors like GaAs or InP for photodetection, SiNW is a motivating material based on the matured silicon industry in which the Si‐based CMOS integration process has already been established.^204^ SiNW photodetectors, such as p–n junctions, p–i–n photodiodes and phototransistors, have been developed to reduce the operational voltage and enhance the photosensitivity and speed. To enhance the light‐detecting performance, many research groups have studied the characteristics of the heterostructure combining Si with metal, semiconductors, or organic materials and changed device structures by the demonstration of specific growing processes.

In this section, we report and discuss the current status, advances, and future in the area of photodetecting devices including photovoltaics using SiNW.

### 4.1 Optoelectronic Properties of SiNW Photoconductors

Due to the unique and controversial optoelectronic properties of 1D nanostructures, researchers actively contribute to the better understanding of the optical response of SiNWs and respective device applications.^205^ Unlike bulk structure, optoelectronics of nanostructures is inherently influenced by the surfacial or interfacial traps and quantum effects. In this section, we summarize most important studies related to the size‐induced effects in the optoelectronic properties of SiNW.

In 2005, photocurrent in an individual SiNW FET was measured by Ahn et al. using optical scanning measurement^206^ thanks to the development of nanowire synthesis technique (a vapor–liquid–solid mechanism) with controllable diameter.^82^ This study shows that non‐Ohmic contact properties play a crucial role in the light‐induced responses, which is shown by scanning images that reveal the local energy band profile near the contacts. This technique is especially powerful to probe the behavior of the local electrostatic potentials. In addition, photocurrent is depending on gate bias which implies the direct implantation of nanowire‐based optics in the conventional CMOS technology.

**Figure 13:**
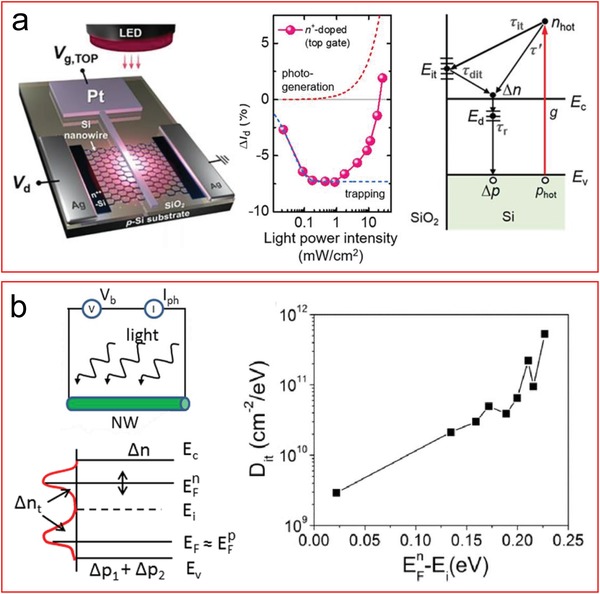
Effect of Interfacial trapping of electrons in SiNWs. a) The negative photoconductance of heavily doped SiNW FETs and the trapping phenomena. Adapted with permission.^208^ Copyright 2017, ACS Publications. b) The photoinduced current change shows trapping could be the major source of the extraction of interfacial trap density using photoconductance analysis of SiNWs. Adapted with permission.^213^ Copyright 2015, American Institute of Physics (AIP Publishing).

The first report of the doping effect in photoconductance of SiNWs was published in 2006.^207^ The nanowires were synthesized by thermal CVD method. Photoresponses of undoped, n‐, and p‐type SiNWs were investigated under the light illumination of UV and near‐infrared region. Here, undoped devices showed high sensitivity in NIR light illumination, but n‐ and p‐type devices did not show the red light responses. However, in this study, the correlation between the doping profile and the photoconductance was not investigated, and comparably slow photoresponse of doped NWs implies that numerous defects or trapping cites could exist in the synthesized NWs. The photoresponse of the doped SiNWs was clarified in the studies of Baek et al. in 2017^208^ (**Figure** **13**a). In this study, SiNW FETs were fabricated by the modern top‐down CMOS process. It was demonstrated that inevitable trap states produced by dopants and interfaces critically inhibit the photoconductance by reducing the mobile charges. Therefore, negative photoconductance can be induced even under visible light illumination with low power intensity. Also, this study shows that the photoelectron trapping by dopant states becomes dominant under the red light illumination because of the indirect energy band structure of silicon, which can also explain the lack of red light sensitivity of doped SiNWs in the previous study in 2006.^207^

**Figure 14:**
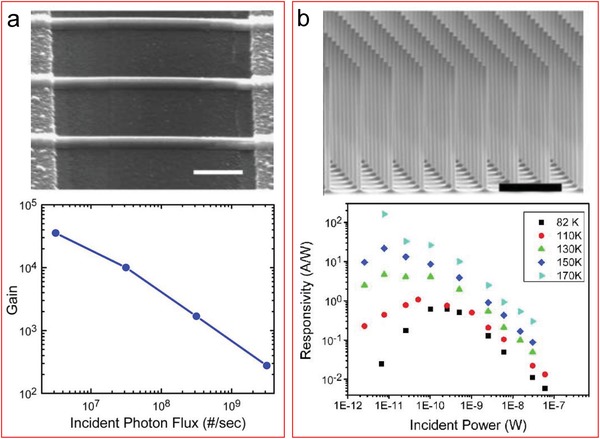
Phototransitive characteristics of SiNWs. a) The planar multi‐SiNW photodetector showing phototransitive gain which is dependent on the photon flux. Adapted with permission.^209^ Copyright 2008, AIP Publishing. b) Vertically etched SiNW phototransistor and the responsivity under the infra‐red light illumination. Adapted with permission.^212^ Copyright 2010, ACS Publications.

As shown in the previous studies, the surface (or interface) trap states play a dominant role in optoelectronic properties in nanowire devices which is comparably negligible in bulk Si devices. Figure 13b shows that trapped electrons by surface trap states can act as a gate bias of the SiNW that enhances the photoconductive characteristics.^208^ Zhang et al. demonstrated that SiNW photodetectors show large phototransitive gain defined as *G* = *I*/*eF* (where *I* is the photocurrent, *e* is the elementary charge, and *F* is the photon flux per second)^209^ (**Figure** **14**a). Both planar and vertical SiNW photodetectors fabricated with the top‐down approach had very high gain, and vertical SiNW showed significantly improved fill factor (FF, the ratio of a light‐sensitive area to total area) due to the increased effective coupling efficiency by waveguided light in the nanowire. Kelzenberg et al. demonstrated the enhancement of light absorbance characteristics of the vertical SiNW arrays.^210^ Similarly, amorphous Si nanocone arrays also showed improved absorption characteristics.^211^ This vertical NW photodetector could detect low‐power infrared light (1550 nm), as well as ultralow‐power visible light^212^ (Figure 14b). Those sub‐bandgap detection was because of the band‐to‐surface photoinduced carrier generation in the nanostructure. In 2015, Dan suggested the method to calculate the density of trap states by measuring the frequency‐dependent photoconductance^213^ (Figure 13b). He showed that photoconductance gain is due to the gating effect depending on surface trapped photogenerated carriers.

In addition, Si crystallinity‐related studies have been published during the last years. Shiri et al. investigated the photoinduced carrier dynamics of strained intrinsic SiNW FET with 1.7 nm diameter using Monte Carlo simulations.^214^ The simulation result shows the effects of multiphonon carrier scattering on the photoinduced carriers. Dhara et al. studied circular photogalvanic effect (CPGE, the magnitude and polarity of photocurrent depend on the chirality of polarized optical excitation) of SiNW which does not occur in bulk Si.^215^ Recently, Yi et al. developed a bioinspired subwavelength photodetection pixel that can measure both the intensity and incident angle of light using two closely spaced SiNWs.^216^ Optically coupled two NWs act as resonators that scatter light and are able to determine the incident angle.

Taking the well‐known optical properties of SiNWs into consideration, flexible and partially transparent SiNW photodetectors were fabricated on a polyethyleneterephthalate substrate.^217^ The devices reached stable photoswitching characteristics after hundreds of bending cycles.

### 4.2 SiNW Photodiodes

The traditional photodetector structure is a two‐terminal junction diode. The goal of early studies of NW photodiodes was to overcome low light sensitivity originated from the nanometer scale. In order to amplify the current signal, the avalanche photodiodes were fabricated by making the junction like p–n^218^ or p–i–n SiNW^219^ that are operated at bias close to the breakdown.

To improve the photodetecting performance like FF, researchers have been developing vertical SiNW structures and implementation techniques which have become a popular trend in photodiodes research up to now.^220^, ^221^, ^222^ Vertical SiNW arrays can be fabricated using both, bottom‐up and top‐down methods. Vertically grown SiNW uses template‐assisted chemical etching technique.^220^ To form metal–insulator–semiconductor (MIS) photodiodes and fix the vertical NW arrays, the SiNWs are filled with indium–tin oxide (ITO).^209^, ^220^ On the other side, current studies tend to use a sophisticated top‐down Si etching technique using electron beam lithography.^209^, ^212^, ^221^, ^222^ Zhou et al. developed a vertical core–shell p–n diode using intrinsic properties of Si.^221^ Thanks to the various quantum effects in NWs, the devices overcome the limits of intrinsic Si, such as sub‐bandgap infrared absorption. Also, vertical p–i–n Si photodetectors are reported as color image sensors.^222^ Each NW is used for one pixel which can have various spectral responsivity depending on its radius.

### 4.3 Hybrid SiNW Photodetectors

SiNW heterostructures have been widely studied to improve the photodetecting performance. The main photoconductive material is SiNW which is supported by cooperating materials such as Au,^223^, ^224^, ^225^, ^226^, ^227^ graphene,^226^, ^228^ and quantum dots (QDs).^229^ SiNWs are decorated with plasmonic Au nanoparticles to enhance the polarization sensitivity^223^ or Si Raman scattering and photocurrent.^225^ Au nanoparticles also generate photocurrent transient effect with SiNW FET.^227^ In an optical manner, Au film‐deposited SiNW heterostructure was fabricated to tune optical modes by controlling absorption and scattering in gold and Si nanostructure.^224^ These studies imply that surface plasmon resonances in Au can increase photocurrent in SiNW depending on the light polarization.

Graphene is used to widen the detectable spectrum region. An NIR photodetector was fabricated with vertical SiNW arrays that were covered with Au nanoparticles decorated graphene on the top of the vertical NWs.^226^ This device uses surface plasmon and light trapping of Au nanoparticles and graphene that supports strong absorption in NIR range. Also, visible‐to‐terahertz range detection was observed using reduced graphene oxide–SiNW heterostructure.^228^ Similarly, a SiNW device with CdTe QDs showed an improvement of photocurrent in UV range.^229^

In addition, metal/semiconductor vertical core–shell photodetectors such as Ag/Si^230^ or Cu/Si^231^ have been studied. Ramadurgam and Yang simulated semiconductor–metal–semiconductor core–multishell nanowire structure to offer a high visible range tenability using plasmon hybridization with low loss, isotropic, and polarization‐dependent negative‐index metamaterial properties.^230^ Cu nanofilm and Si heterostructures improve the device to detect NIR light with fast and high on/off switching characteristics.^231^ Similarly perovskite (CH_3_NH_3_PbI_3_)/SiNW core–shell photodetector could detect NIR light.^232^ Wu and co‐workers fabricated a Ni_2_Si/Pt_2_SiNW axial heterostructure with the in situ transmission electron microscopy (TEM) observation. Pt/Ni/Si ternary NW heterostructure shows the excellent infrared‐sensing property, which is not shown with the Ni_2_Si/Pt_2_SiNW heterostructure. Those inorganic heterostructures show the ability to tune and broaden the detecting spectra of light by coating SiNWs with a plasmonic metal film or nanoparticles, which can overcome the limitation of the bandgap of SiNWs.

**Figure 15:**
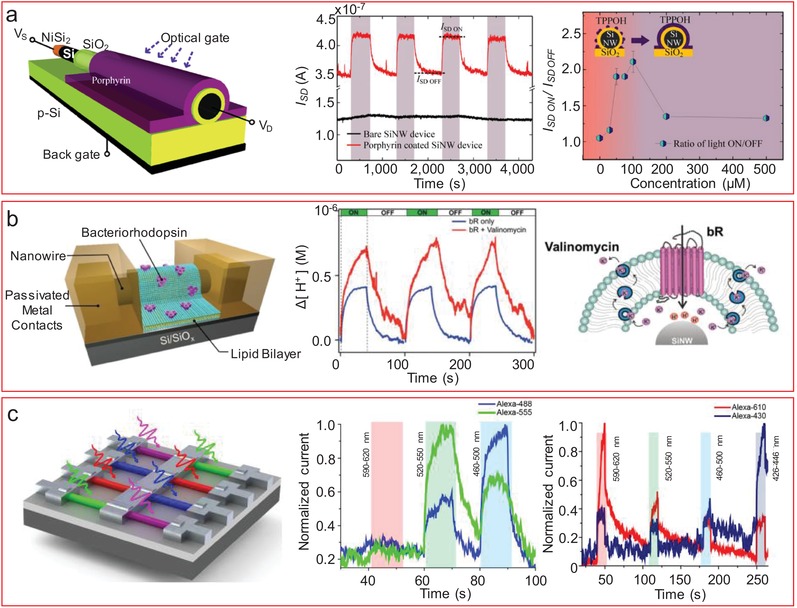
Organic–inorganic hybrid SiNW photodetectors. a) Optical gate of organic photosensitive molecule (porphyrin)‐coated SiNW FETs. The hybrid shows stronger light‐induced current switching characteristics. The current on/off ratio depends on the thickness of the film. Adapted with permission.^3^ Copyright 2015, Springer. b) Bioinspired gate using proteins within lipid bilayer of the SiNWFETs. Adapted with permission.^10^ Copyright 2015, Wiley‐VCH. The light modulates the charge transporting of proteins. c) Molecular embedded SiNW FETs which detect multiple wavelengths. Adapted with permission.^236^ Copyright 2018, ACS Publications.

Meanwhile, many groups have developed organic and SiNW hybrid photodetectors to exploit the excellent photosensitive properties of organic molecules as shown in **Figure** **15**. Among various organic materials, porphyrin had the greatest attention, since it is the light‐trapping molecule in plant photosynthesis. The first porphyrin‐coated SiNW FET was studied in 2007.^74^ Unlike other SiNW heterostructures that showed broad absorption spectra, photocurrent switching of porphyrin‐coated devices strongly follow the two distinct peaks (Soret‐ and Q‐bands) in the absorption spectrum of porphyrin. Also, direct charge‐transfer characteristics from porphyrin to SiNW was studied using a covalent bonding of porphyrin.^233^ In this study, photocurrent was measured in liquid like on the conventional bioFETs, and the highest photocurrent was observed in the NIR region.

The next featured application is a complementary logic gate consisting of n‐ and p‐type SiNW FETs covered with porphyrin.^234^ Porphyrins are acting as an acceptor with n‐channel and as a donor with p‐channel devices that make different photocurrent switching direction. This porphyrin‐coated inverter is turned on and off by light illumination. The same group studied subsequently the optical and electrical charging and discharging of porphyrin‐coated SiNW FETs.^235^ In 2015, the optical gating effect of porphyrin‐covered SiNW FETs was demonstrated^3^ (Figure 13a). Unlike other groups that used direct electron tunneling from the molecules to the nanowire, this study used the field‐effect of the light‐induced charge in the porphyrin shell wrapped on the SiO_2_ dielectric layer to realize the optical gate literally.

Apart from porphyrin as a bioinspired gate structure, a light‐gated membrane protein residing within a lipid bilayer, of SiNW FETs was demonstrated^10^ (Figure 15b). The microbial proton pump bacteriorhodopsin formed channels in the lipid bilayer, and coupled light‐driven proton transport to up‐ and downregulate the device output by modulating the membrane potential and ion permeability of membrane. Recently, Lefler et al. reported molecularly embedded SiNW FETs which could detect different wavelengths (red–green–blue–violet) without color‐specific filters^236^ (Figure 15c). Various Alexa fluorophores were functionalized on each NW device, which were highly stable in wet condition.

The publications about hybrid SiNW photodetectors just started to increase since 2011. The hybrid SiNW devices are promising elements as the next‐generation photodetectors, due to the various functions of combined photosensitive materials and large degree of freedom in material selection. The large surface‐to‐volume ratio of nanowires can maximize the surface charging effect induced from the outer coated material. It is also attractive to device designers who can imagine many varieties in applications with diverse optical‐sensitive materials from small molecules to polymers or plasmonic metals. However, organic‐coupled devices still suffer from the slow switching speed and instability in variable environmental conditions.

### 4.4 Solar Cells

PV cells, i.e., solar cells, have been explosively growing over the past decade with urgent requirement to seek the sustainable energy source in accordance with the environmental and commercial requirement for the reduction of CO_2_ emission.^237^ p–n junctions absorb photons to generate mobile electron and hole pairs which are collected by the electrodes depending on energy band structure. Although the junction structures of solar cells and photodiodes are equal, the main functions are different. Since the solar cells are energy‐conversion devices, the light to power conversion efficiency (PCE) becomes the most important factor in the solar cell. Si, especially crystalline Si, is the most popular material in commercial PV market due to their high efficiency and stability as well as material abundance and well‐defined processing technology.^238^, ^239^ Among various Si‐based structures from bulk, thin film to nanocrystals, 1D NW structure has several advantages: i) strong absorption characteristics by light trapping in the NW by multiple scattering events, and ii) short carrier path length in radial junction. Two types of junction structure such as axial and radial junctions (i.e., core–shell) are mainly used for fabricating NW solar cells.^240^ Since this area is very actively and rapidly growing, the solar cell research community shares the information of highest power efficiency every year^241^ and publishes review articles frequently.^237^, ^238^, ^239^, ^242^ Therefore, in this session, we will focus on the general working principle and structures of SiNW solar cell to understand the functional advantages of hybrid devices in the following section.

In 2005, the first SiNW PV application was reported.^243^ The vertical SiNW arrays were prepared with chemical‐etching technique based on a galvanic displacement reaction. Because of the advantages of vertical NWs for optical detection (as explained in previous sections), most of NW solar cells have vertical structure. The SiNWs show higher absorption compared to solid Si film due to the reduced optical reflection.^244^ The PV property of a single SiNW having rectifying contacts was investigated regarding the resistivity and diffusion length.^245^ This study suggests that device geometry can be the key factor of the optical absorption and transmission due to the interference of the front and back surfaces of the nanowires. Also, SiNWs show the optical antenna effect that can be used for photon management (e.g., trapping photons in a broad bandwidth) and enhance the optical absorption.^246^ As mentioned in a previous study, the geometry of nanowires such as the cross‐sectional structures as well as a diameter and the space between nanowires are important factors to control the absorption efficiency.

The PV behavior is improved in junction structures.^240^, ^247^, ^248^ Tian et al. fabricated core–shell p–i–n SiNWs to achieve high energy conversion efficiency.^240^ The advantage of the core–shell structure is the short distance of the carrier collection by using radial carrier separation instead of axial direction, and the high efficiency without bulk recombination. On the other hand, tandem p–i–n+–p+–i–n SiNW PV elements have been compared with single p–i–n elements modulated with the axial direction.^247^ The tandem structure shows larger open‐circuit voltage than the single junction device. This study shows that the junction design plays an important role to improve the performance of the solar cell. In 2012, hexagonal cross‐sectional structures of core–shell p–i–n SiNWs were investigated by determined external quantum efficiency (EQE) spectra and absorption mode profiles in the nanowire depending on diameters.^248^ The number of resonant absorption modes is increasing with the size of the nanowire. Since the resonant modes are excited in high‐symmetric structure, rectangular cross‐sectional nanowires show enhanced EQE value in longer light wavelength. This study implies that the morphology of the nanowire is critical to modulate the absorption. Adachi et al. reported that disordered core–shell nanowire devices exhibit enhanced short‐circuit current in both amorphous and crystalline Si shells compared to corresponding planar devices.^249^ SiNW solar cell studies, using intrinsic properties of Si, have mainly shown the fundamental NW‐based solar cell processing technique and the improved performances by controlling doping, junction design, or morphology of NWs.

### 4.5 Hybrid Solar Cells

In addition to the control of doping and geometry of SiNWs, recent studies have stepped toward hybrid SiNW heterojunctions decorated with optically functional materials from plasmonic metal nanoparticles, QDs, to organic materials. The inorganic hybrid materials combining to SiNWs are similar to hybrid photodetectors, but the organic materials tend to be conductive polymers to collect photoinduced electrons.

By electrical coupling the auxiliary nanomaterials and SiNWs, one can tune the absorption efficiency of the device. Metal nanoparticles, e.g., Ag nanocrystals, were decorated on core–shell SiNW p–n junctions to exploit the localized surface plasmons.^250^ Interestingly, the absorption in the hybrid SiNW structure increases under the special wavelength range of light. The increased (or decreased) peaks are originated from the dipolar and quadrupolar surface plasmon resonances of nanocrystals and resonance modification by NW itself. Liu et al. fabricated organic material combined with Si NW/Ag nanoparticle PV cell to improve NIR efficiency by plasmonic hot‐electron injection.^251^ QDs are also interesting materials to be decorated on the NW solar cells.^252^, ^253^, ^254^ There are various choices in QD materials, for instance, PbS^252^ or carbon^253^ that are used as hole‐transporting layers or Si nanocrystals^254^ as effective photon‐harvesting materials. This structure reduces recombination and enhances optical absorption due to the large interfacial area and a short traveling distance for photogenerated minority carriers to the collector. The thickness of the QD shell is an important factor to increase the PV performance in the heterostructure that controls the hole transporting and light blockage. In addition, perovskite can increase the absorption efficiency when combined with SiNW arrays based on its large absorption coefficient.^255^ Finally, metal/SiNW composites (metal = Pt, Ru, Ir, or Rh) have been tested for solar cell applications. Among many metals, Pt/SiNW composite shows the best PCE and high electrochemical stability.^256^

The other branch of the hybrid solar cells is hybrid organic/SiNW heterojunction devices that have shown outstanding properties, such as comparably high PCE among various SiNW‐based solar cells, as well as low‐cost and low‐temperature processing. The most commonly used organic material forming a heterostructure on a (typically n‐type crystalline) SiNW is poly(3,4‐ethylene dioxythiophene):poly(styrenesulfonate) (PEDOT:PSS) which is a transparent and conductive polymer playing a key role as hole‐/electron‐transporting/emitter layer. This organic hybrid structure replaces conventional Si‐based p–n junction. Table 1 shows the various organic junction materials and PV characteristics of prominent hybrid devices. The PCE of hybrid solar cells is in the range of 10–16.1% in last 5 years. The notable point is that the additional hybrid layer or materials, apart from SiNW and PEDOT:PSS, enhance the efficiency by supporting the electron/hole collection.^72^, ^251^, ^257^, ^258^, ^259^, ^260^, ^261^, ^262^, ^263^, ^264^, ^265^

**Figure 16:**
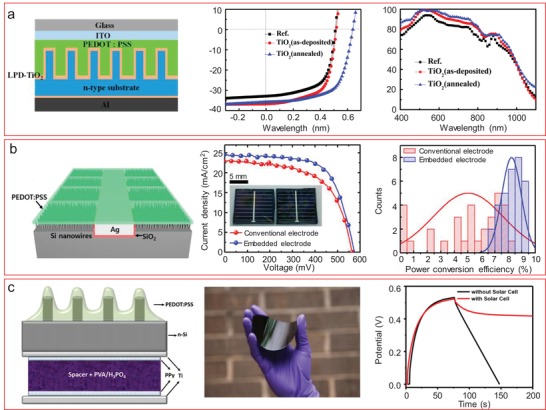
Hybrid organic PEDOT:PSS/SiNW heterostructures of solar cells. a) Organic/SiNW hybrid solar cell with inorganic conformal layer. The current–voltage characteristics and EQE of the devices under illumination. Adapted with permission.^267^ Copyright 2016, ACS Publications. b) Hybrid SiNW solar cell using embedded Ag electrode. Current density–voltage curve and efficiency distribution curves show the increased performance of the solar cell with the embedded electrode. Adapted with permission.^268^ Copyright 2017, ACS Publications. c) Polymer hybrid solar cell supercapacitor. The supercapacitor is fabricable on the flexible wafer. The inset curves show charging and discharging of the flexible super capacitor with and without solar cell. Adapted with permission.^12^ Copyright 2017, ACS Publications.

The effective PV cells reaching near 13% efficiency are including additional organic layers like 1,1‐bis[(di‐4‐tolylamino)phenyl]cyclohexane (TAPC)^260^ or 1,3‐bis(2‐(4‐*tert*‐butylphenyl)‐1,3,4‐oxadiazol‐5‐yl) benzene (OXD‐7).^264^ TAPC, positioned in between the PEDOT:PSS and the SiNW, suppresses the interface recombination and strong oxidation reaction at the interface. Also, rear surface processing with OXD‐7 has enhanced PCE by preventing the large rear surface recombination. Park et al. showed that an Au mesh electrode improves PCE (13.2%) by optimizing the line pitch of the mesh electrode which determines optical transmission into the active layer.^266^ A liquid‐phase deposited titanium dioxide (LPD‐TiO_2_) interlayer between SiNW and PEDOT:PSS dramatically increases PCE (14.7%)^267^ (**Figure** **16**a). The TiO_2_ layer helps the PEDOT:PSS flow into space between NWs. Because of the high hole‐tunneling rate through the TiO_2_, high carrier transport was observed.

There are several studies that obtain high efficiency (e.g., 13.36%,^257^ 16.2%,^268^ and 14.08%^269^) in PEDOT:PSS/SiNW hybrid junction without any additional material. Subramani et al. used a low‐pressure vacuum‐assisted coating method to make better polymer coverage and reducing the surface defects by chemical polishing etching of the Si surface.^257^ Hybrid solar cells using embedded Ag electrode showed the best efficiency (16.2%) among our search database^268^ (Figure 16b). This special design can avoid the localized short circuit, which degrades the PV performance in the solar cell with a conventional electrode design. Duan et al. applied tetramethyl ammonium hydroxide (TMAH) treatment which leads to smooth SiNW surface with a balanced light‐trapping property and surface defects.^269^ These studies imply that the interfacial quality of the SiNW is critical to achieve the high efficiency.

The fabrication and performance of flexible photovoltaic cells were also recently presented.^12^, ^251^, ^263^ For the flexible devices, vertical nanowire arrays are fabricated using metal‐assisted chemical etching on the ultrathin Si membranes or on normal Si wafer, which is thinning to sub‐10 μm thickness using inductively coupled plasma‐reactive ion etching (ICP‐RIE). Up to now, the efficiency is comparably low because of nanowire morphology and a worse contact of the NW heterostructure to front and rear electrodes.

The structural and material diversity of SiNW‐based solar cells allows increasing PCE. Although it is not yet efficient enough to be used for commercial purposes, we expect that various applications will be possible by novel hybrid material integration. An overview of the photovoltaic characteristics of various recent hybrid solar cells is shown in **Table** **3**.

**Table 3:** Photovoltaic characteristics of various hybrid solar cells

Device material	*V*_OC_	*J*_SC_ [mA cm^−2^]	FF [%]	PCE [%]
In:Ga/n‐SiNW/spiro‐OMeTAD/Cu/PEDOT: PSS/Cu:Ag^72^	0.527	31.3	58.8	9.70
Ti/Ag/n‐SiNW/PEDOT:PSS/ITO^259^	0.532	24.24	65.14	8.40
In:Ga/n‐SiNW/P3HT/Cu^290^	0.457	37.6	54	9.2
Al/n‐SiNW/TAPC/PEDOT:PSS/Ag^260^	0.54 ± 0.005	34.81 ± 0.05	67.08 ± 2.27	12.54 ± 0.49
Al/n‐SiNW/PEDOT:PSS/Ag^261^	0.52	34.46	64.06	11.48
Al/n‐SiNW/PEDOT:PSS/Ag^262^	0.465	30.65	65	9.3
Ni/Ag/AlO*_x_*(AgNPs)/n‐SiNW/PEDOT:PSS/Ag (flexible)^263^	0.545	18.50	65.5	6.62
Al/OXD‐7/n‐SiNW/PEDOT:PSS/Ag^264^	0.557	33.0	69.9	12.9
Ti/Pd/Ag/n‐SiNW(SiO*_x_* shell)/PEDOT:PSS/Ag^265^	0.58 ± 0.01	30.8 ± 0.7	67.7 ± 1.4	12.2 ± 0.2
Al/Si/SiNW/PEDOT:PSS;Ag^289^	0.614	30.42	70	13.11
n‐SiNW/Al_2_O_3_/PEDOT:PSS/Au mesh^266^	0.539	36	67.8	13.2
Ag/Al/n‐SiNW/AgNPs/PEDOT:PSS/ITO (flexible)^251^	0.45	19.10	46.4	3.92
Ti/Ag/n‐SiNW/PEDOT:PSS/glass/ITO^257^	0.528	35.67	70.94	13.36
Al/n‐SiNW/LPD‐TiO_2_/PEDOT:PSS/ITO/glass^267^	0.63	35.91	65	14.70
[Ti/PPy/Spacer+PVA/H_4_PO_4_/PPy/Ti]/n‐SiNW/PEDOT:PSS/Ag (flexible)^12^	0.59	31.38	72	13.39 (storage efficiency: 10.5%)
n‐SiNW/embedded Ag/PEDOT:PSS^268^	0.607	34.0	78.3	16.1
Al/SiNW/PEDOT:PSS/Ag grids^269^	0.632	31.53	71	14.08

spiro‐OMeTAD: *N* ^2^,*N* ^2^,*N* ^2^′,*N* ^2^′,*N* ^7^,*N* ^7^,*N* ^7^′,*N* ^7^′‐octakis(4‐methoxyphenyl)‐9,9′‐spirobi[9H‐fluorene]‐2,2′,7,7′‐tetramine; PPy: Polypyrrole; PVA: Polyvinyl alcohol.

## 5 Biologically Inspired Hybrid Devices

The ultimate dream of the research and development community working in the area of artificial intelligence (AI) is to develop the computing machine that would resemble the functions of the brain.^270^ The brain, a natural computing machine, has a great ability to predict, analyze, and infer links among the events, which are difficult tasks for nowadays computers. The brain consists of around 100 billion neurons in which simultaneous processing and storing information take place via complex 10 trillion synaptic connections^271^ In this context, brain‐inspired neuromorphic architecture has been proposed to mimic the neuron and synaptic behaviors and phenomena in the neural network. Recently, the several inspiring developments related to artificial neurons and synapses have been published. Although memristive memory cells are mainly used for artificial synapses, many synaptic or neuron‐like transistors using either memristive channel^13^, ^272^, ^273^, ^274^, ^275^, ^276^, ^277^ or dielectric materials^278^, ^279^, ^280^, ^281^, ^282^, ^283^, ^284^ have been reported.

**Figure 17:**
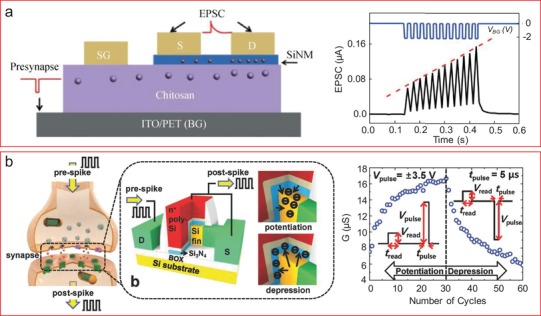
Artificial synapses using Si nanostructures. a) Si nanomembrane (SiNM) transistors gated by chitosan membrane. Ionic excitatory postsynaptic current (EPSC) response to the voltage pulses train applied on the back gate (BG). Adapted with permission.^282^ Copyright 2018, Wiley‐VCH. b) Recoverable synapse devices using Si finFET. The biological synapse is mimicked using Si FinFET with the electron trajectories during the potentiation and depression. Conductance modulation by the applied pulse cycles. Adapted with permission.^13^ Copyright 2017, IOP Publishing.

SiNWs represent an excellent building block to form the brain‐inspired devices, e.g., for transistor integration combining with memristive dielectric material, which could be mobile‐ion‐doped oxide or gel structure. Lai et al. provided the initial concept of synaptic transistor using Si MOS transistors.^278^ A conjugated polymer layer of poly[2‐methoxy‐5‐(2′‐ethylhexyloxy)‐*p*‐phenylene vinylene] (MEH‐PPV) and an ionic conductive layer of RbAg_4_I_5_ were stacked on the SiO_2_ insulting layer. Voltage spikes on the polymer layer transient ionic fluxes and modify nonvolatile ion storage, which provide synaptic plasticity. In 2017, Si nanomembrane transistors gated by chitosan membrane^282^ (**Figure** **17**a). Proton migration in the chitosan is triggered by the gate voltage pulses and causes synaptic plasticity.

On the other hand, Hur et al. developed synaptic transistors using Si‐based Fin‐channel with poly‐Si/SiO_2_/Si_3_N_4_ gate stack^13^ (Figure 17b). The synaptic behavior was generated by electron trapping in the gate layer, which is responsible for channel depletion. This trapping is a reversible process and can be recovered to an initial state by an annealing process. This study shows that the electron‐trapping mechanisms in Si MOSFETs without taking into account any organic‐based ionic layers can be the dominant driving force to induce the synaptic plasticity in the devices. Similarly, the synaptic transistor using water molecular trapping on the carbon nanotube surface which induces the synaptic plasticity was reported as well.^276^

Up to now, SiNW‐based artificial neurons or synapses are not reported up to our knowledge. However, since the transistor has a striking structural similarity to a neuron,^280^ such as signal transfer through main channel and threshold‐based voltage control, it is expected that SiNW FETs will provide stable electronic performance to emulate biological behavior. For the realization, hybrid structure combining SiNWs and a material which can mimic the ion dynamics of the neuron cell membrane would be necessary. The possible candidates could be resistive switching ones using a conductive ion bridge^285^ or novel artificial bioinspired materials containing ions. Also, the size effect of NW like interfacial trapping has arisen as the new issues or the solution to build novel biomimicking devices.

## 6 Conclusions and Outlook

Nanowires as a part of silicon technology are a promising building block of the devices and circuits with a demonstrated prominent future. Multiple alternative fabrication and configuration strategies can adapt to specific needs, while the standard procedures can result in the development of arrays with thousands of individually addressable devices in a tiny circuit integrated with other functionalities.

Major part of the published results on SiNW‐based devices are dedicated to their performance as (bio‐)sensors and are focused on the description of the phenomena/effects, caused by external stimuli (e.g., gas, liquid, and biological or chemical species) on the channel conductance. As described above, SiNW FETs are already capable of detecting very small concentrations of biomolecules in complex samples following the appropriate techniques, and this can be a strong and convenient advance pushing the biomedical field to reduce the workload of clinics and hospitals. However, in order to reach the aforementioned application phases, nanowire‐based sensorics should grow up beyond the device level and evolve into the system level, where individual sensor elements are embedded into the properly designed conditional electronics network for data collection, filtering, amplification, and further transfer. Complete biomarker panels, mutations, or genetic diseases along the whole genome, infectious diseases, and many other targets can be the objective of a single chip.

However, one inevitable hurdle to solve is how to give a different functionality to each of the sensors of such high‐density chip considering the small separation between them. Localized Joule heating^286^, ^287^ can be a possible solution, where nanowires are coated with a protective polymer and they can be one by one uncoated by applying an electrical bias to produce localized ablation of the polymer followed by the functionalization, only on the selected nanowire.

Further, SiNW photodetectors and their physics have been studied adapting various inorganic or organic material and structural combination.^288^, ^289^ Those hybrid systems can enhance the photodetection efficiency and provide specific functionalities like wavelength selectivity or photoreactivity depending on molecular dynamics, which are hard to realize using bare SiNWs. Although Si already accounts for the most significant portion of the solar cell market, the SiNWs are attractive alternative because of the large surface area‐to‐volume ratio. Additionally, by utilizing the properties of organic materials, solar cells with new properties including flexibility can be produced.

The bioinspired SiNW devices are a comparably new, but extremely fast‐growing, field. For these devices, a hybrid property is fundamental to emulate the biological phenomena based on ion transport. Although there are not many silicon‐based devices available yet, it is anticipated that a variety of applications will be published that take advantage of the electrical characteristics of Si. As the development of recent neuromorphic cells began with the study of emerging memory devices, Si‐based memory that utilizes the trap characteristics will be able to be fused.

The major challenges that we need to overcome include i) the instability of some organic materials that are difficult to maintain and persist for a long time, and ii) interface trap states caused by heterostructures which could slow down the overall speed. Therefore, it is necessary to search for new organic or inorganic materials to solve the problems and further attempts to study, or actively use, the physics of interface traps. In this respect, the hybrid device architecture is a hot research topic that opens a broad range of new possibilities that can be added to the spectrum of already demonstrated existing strong technology^290^ to obtain extended functionalities and create new ones, without the need of replacing the silicon as base material.

## Conflict of Interest

The authors declare no conflict of interest.
